# Intrinsically disordered electronegative clusters improve stability and binding specificity of RNA-binding proteins

**DOI:** 10.1016/j.jbc.2021.100945

**Published:** 2021-07-09

**Authors:** Steve Zaharias, Zihan Zhang, Kenneth Davis, Talia Fargason, Derek Cashman, Tao Yu, Jun Zhang

**Affiliations:** 1 Department of Chemistry, College of Arts and Sciences, University of Alabama at Birmingham, Birmingham, Alabama, USA; 2 Department of Chemistry, Tennessee Technological University, Cookeville, Tennessee, USA; 3 Department of Chemistry, University of North Dakota, Grand Forks, North Dakota, USA

**Keywords:** electronegative clusters, intrinsically disordered proteins, acidic patch, poly-D/E, RNA-binding proteins, low-complexity regions, CSPs, chemical shift perturbations, DBPs, DNA-binding proteins, DSC, differential scanning calorimetry, ENCs, electronegative clusters, FP, fluorescence polarization, non-NBPs, non–nucleic acid–binding proteins, PRE, paramagnetic relaxation enhancement, RBPs, RNA-binding proteins, RRM, RNA-recognition motif, SRSF3, Ser/Arg-rich splicing factor 3, TDP-43, TAR DNA binding protein 43, TDP-43 RRM2, second RRM domain of TDP-43, ΔPRE, PRE difference

## Abstract

RNA-binding proteins play crucial roles in various cellular functions and contain abundant disordered protein regions. The disordered regions in RNA-binding proteins are rich in repetitive sequences, such as poly-K/R, poly-N/Q, poly-A, and poly-G residues. Our bioinformatic analysis identified a largely neglected repetitive sequence family we define as electronegative clusters (ENCs) that contain acidic residues and/or phosphorylation sites. The abundance and length of ENCs exceed other known repetitive sequences. Despite their abundance, the functions of ENCs in RNA-binding proteins are still elusive. To investigate the impacts of ENCs on protein stability, RNA-binding affinity, and specificity, we selected one RNA-binding protein, the ribosomal biogenesis factor 15 (Nop15), as a model. We found that the Nop15 ENC increases protein stability and inhibits nonspecific RNA binding, but minimally interferes with specific RNA binding. To investigate the effect of ENCs on sequence specificity of RNA binding, we grafted an ENC to another RNA-binding protein, Ser/Arg-rich splicing factor 3. Using RNA Bind-n-Seq, we found that the engineered ENC inhibits disparate RNA motifs differently, instead of weakening all RNA motifs to the same extent. The motif site directly involved in electrostatic interaction is more susceptible to the ENC inhibition. These results suggest that one of functions of ENCs is to regulate RNA binding *via* electrostatic interaction. This is consistent with our finding that ENCs are also overrepresented in DNA-binding proteins, whereas underrepresented in halophiles, in which nonspecific nucleic acid binding is inhibited by high concentrations of salts.

Intrinsically disordered protein regions constitute a third of the human genome (1, 2). They are more prevalent in RNA-binding proteins (RBPs), accounting for 50% of the RNA-binding proteome (3). Despite the lack of persistent structure, disordered regions possess posttranslational modification sites (4) and protein-binding motifs (5, 6). These features enable intrinsically disordered proteins to play indispensable roles in cellular signaling and regulation (5). Therefore, mutation of disordered regions frequently results in dysregulation of the involved biological functions or pathological protein aggregation (7).

The disordered regions in RBPs feature repetitive sequences, such as poly-A, poly-G, poly-N/Q, and poly-K/R residues (3, 7, 8). Poly-A, poly-G, and poly-N/Q are involved in phase separation (7, 8). For example, the poly-Q/N region of TAR DNA binding protein 43 (TDP-43) is responsible for phase separation through mediating intermolecular interactions (9). The length of poly-N/Q regions is critical for their functions, as expansion of poly-Q regions is frequently related to neurodegenerative diseases and various cancers (10). Similarly, extended poly-A regions cause *in vivo* protein aggregation (11, 12). Poly-K/R motifs play a role in RNA binding, RNA folding, and nuclear localization (13, 14, 15, 16, 17, 18, 19, 20, 21, 22). The poly-K/R region of the HIV Tat protein exemplifies its function in prompting both RNA binding and RNA folding *via* electrostatic interactions (23, 24).

A largely neglected family of repetitive sequences is electronegative clusters (ENCs) that contain acidic residues or acidic residues with embedded phosphorylation sites. The functions of ENCs have only been investigated by scattered case studies. For example, Santiago-Frangos and Woodson have found that the acidic tail of the Hfq protein inhibits nonspecific RNA binding and facilitates the recycling of Hfq from an sRNA–mRNA duplex (25, 26, 27). Through Rosetta simulation, they proposed that this inhibition is through interaction between the acidic tail and basic sites on the protein (27). In addition, previous studies on histone pre-mRNA stem-loop–binding protein have shown that hyperphosphorylation of the C-terminal acidic region is essential for high-affinity binding and RNA processing (28, 29). These studies have suggested that ENCs can play important regulatory roles in RBPs, but a systematic examination of their occurrence, an in-depth study of their impacts on RNA-binding specificity, and an experimental characterization of the interaction between ENCs and RNA-binding domains are still lacking.

In this study, we systematically searched for various repetitive sequences in RBPs and revealed a surprising finding that ENCs are more abundant than all other repetitive sequences. We hypothesized that one of ENCs’ functions is to suppress nonspecific RNA binding. To test this hypothesis, we selected yeast Nop15 as a model, as co-occurrence of the ENC with the RNA-recognition motif (RRM) in Nop15 represents the most common situation. Nop15 is essential for large ribosomal subunit biogenesis (30, 31). Nop15 binds to and stabilizes the ITS2 III.A RNA, a stem-loop RNA region that transiently exists during ribosomal biogenesis (31). We found that the ENC stabilizes the neighboring RRM, and the increase in protein stability can be used to measure the dynamic intramolecular interaction between the ENC and the RRM. We further revealed that the Nop15 ENC interacts with the RRM mainly *via* charge interactions. Moreover, we found that the ENC inhibits nonspecific RNA binding, but barely affects specific binding. To further determine the effect of ENCs on sequence specificity of RNA binding, we grafted an ENC to an RRM-bearing protein, Ser/Arg-rich splicing factor 3 (SRSF3). Using RNA Bind-n-Seq, we found that the engineered ENC increases RNA-binding specificity by inhibiting RNA binding. However, the inhibiting effect is discriminating instead of weakening all RNA motifs to the same extent. The site where electrostatic interactions play a dominant role in binding is more susceptible to the ENC inhibition. Our findings may have implication beyond RBPs. We found that ENCs are also overrepresented in DNA-binding proteins (DBPs) relative to non–nucleic acid–binding proteins (non-NBPs). In contrast, ENCs are significantly underrepresented in halophiles, in which the issue of nonspecific RNA binding is addressed by high concentrations of salts.

## Results

### ENCs are the most abundant repetitive sequences in the RBPs’ disordered regions

**Figure 1:**
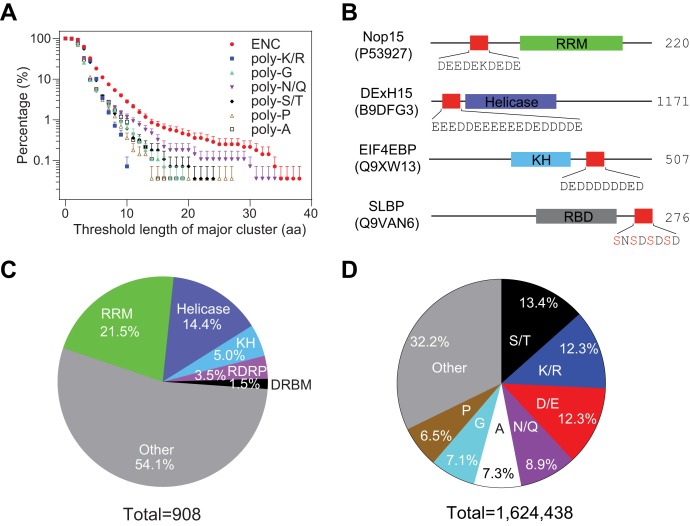
**Electronegative clusters are the most abundant repetitive sequences in RBPs.***A*, percentage of RBPs that have repetitive clusters passing the threshold length. For clarity, only the top halves of the error bars (┬) are shown. *B*, example ENCs in four representative RBPs with UniProt protein ID in the *parentheses*. Ser residues in the SLBP ENC are phosphorylated. The numbers on the right are lengths of example proteins. *C*, the top five RNA-binding domains that occur with ENCs of four amino acids or longer are the RNA-recognition motif (RRM), helicase, K homology (KH), RNA-dependent RNA polymerase (RDRP), and dsRNA-binding motif (DRBM). The category “Other” includes the following 35 RNA-binding domains: AXH, B5, CSD, CP type, DDT, DFDF, Dicer, DZF, exonuclease, FHA, G-patch, HTH, KOW, Macro, MI, MIF4G, Nop, NTF2, PINc, PUM, PUA, PWI, R3H, RAP, reverse transcriptase, RNase, S1, S1-like, S4, SAP, SET, THUMP, tRNA-binding, TROVE, and YTH. *D*, mole percentage of amino acids in RBP disordered regions. ENCs, electronegative clusters; RBPs, RNA-binding proteins; SLBP, stem-loop–binding protein.

To examine the occurrence of repetitive clusters, we analyzed amino acid sequences of 2,783 RBPs that exist at the protein level and contain domain boundary annotations (32, 33). Based on the amino acid side-chain size and polarity, the clusters were grouped as electropositive (poly-K/R), electronegative (poly-D/E or acidic residues with embedded phosphorylation sites, *i.e.*, ENCs), amide containing (poly-N/Q), hydroxyl group containing (poly-S/T), aromatic (poly-F/Y/W), and bulky aliphatic (poly-I/L/V) residues. The amino acids not in the above groups were assumed to form homopolymer clusters. As a protein can possibly have multiple repetitive clusters of different lengths, we define the longest one(s) as the major cluster. Using this definition, we counted the occurrence of major clusters for all aforementioned repetitive sequences. Surprisingly, our systematic search found that the most abundant repetitive clusters in RBPs are ENCs that contain consecutive acidic residues (poly-D/E), or acidic residues with embedded phosphorylation sites (Fig. 1, *A* and *B*, other types of repetitive clusters shown in Fig. S1*A*). A third of RBPs have ENCs of four consecutive amino acids or longer in their disordered protein regions. The longest ENC is found in human nucleolin (UniProt accession number P19338), which has 38 uninterrupted acidic residues. The poly-D/E ENCs are invariable in their negative charges, whereas the ENCs with phosphorylation sites are tunable. For example, phosphorylation of the stem-loop–binding protein ENC enhances its negative charge (Fig. 1*B*). We continued to analyze the types of RNA-binding domains that immediately neighbor ENCs, finding that the top-five RNA-binding domains are the RRM, helicase domains, K homology, RNA-dependent RNA polymerase, and dsRNA-binding motif (Fig. 1*C*). The high co-occurrence of ENCs with the RRM may be partially due to the fact that the RRM is the most abundant RNA-binding domain.

Our search could be biased if RBPs inherently contain a high percentage of acidic residues. To rule out this potential bias, we analyzed the amino acid content of RBPs by their side-chain properties. Our analysis indicated that the mole percentage of acidic residues in disordered regions of RBPs is 12.3%. This is the same as the mole percentage of basic residues and lower than S/T (13.4%) (Fig. 1*D*). We therefore concluded that the high occurrence of ENCs that we observed in RBPs is not due to a general overrepresentation of acidic residues. We further calculated the averaged *p*-values of the occurrence of the major clusters using Monte Carlo simulations, finding that the average *p*-values of ENCs longer than four amino acids are lower than 5% (Fig. S1*B*).

### Intramolecular interactions between ENCs and RNA-binding domains increase protein stability

**Figure 2:**
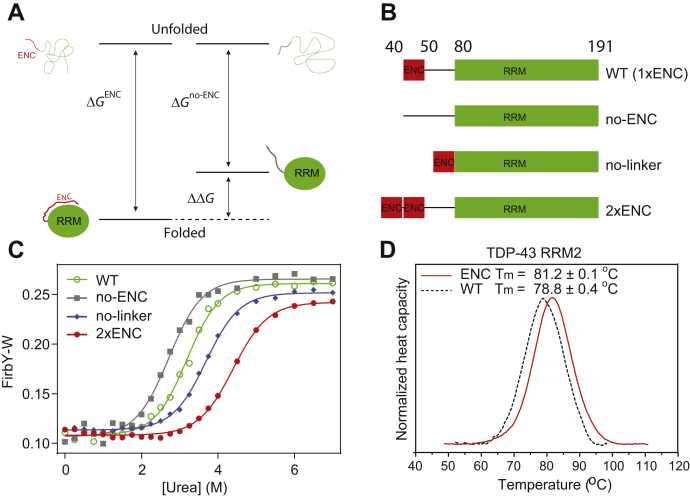
**The Nop15 ENC stabilizes the neighboring RRM.***A*, energetics of intramolecular interactions (*ΔΔG*) between the ENC and RRM is coupled to protein stability. *B*, Nop15 constructs used for protein stability measurements. For the no-ENC construct, the ENC was replaced by a Ser-Gly repetitive sequence of the same length. *C*, urea denaturation profiles of Nop15 constructs measured by FirbY-W. *D*, an artificial ENC can increase stability of TDP-43 RRM2. The melting temperature (Tm) was determined by differential scanning calorimetry. The error was estimated from individual measurements on proteins of three individual preparations. ENCs, electronegative clusters; RRM, RNA-recognition motif; TDP-43 RRM2, second RRM domain of TDP-43.

Intramolecular interactions between ENCs and RNA-binding domains are intuitive because of their opposite charge properties. The challenge is how to quantify the energetics of these dynamic interactions. It is known that intermolecular interactions, that is, ligand binding, increase protein stability and that these energetics (*K*_D_) can be precisely measured by the increase in protein stability (34). Similarly, we propose that intramolecular interactions between ENCs and RNA-binding domains increase protein stability and that these energetics can be measured as illustrated by Figure 2*A*. Here, we assumed that in the unfolded state, there is no interaction between the ENC and the polypeptide of the RNA-binding domain. This assumption is valid under the condition that the RNA-binding domain does not possess long consecutive basic residues. When this condition is not met, the energy of the unfolded state is overestimated and the magnitude of *ΔΔG* is underestimated.

To test our hypothesis, we selected Nop15 as a model protein. Nop15 has a conserved ENC between residues 40 to 44 and 46 to 49, followed by an RRM that binds a stem-loop region of ITS2 III.A RNA. As we found RRMs to be the most common RNA-binding domain to neighbor ENCs and most ENCs only consist of acidic residues, Nop15 is a general model. We created four different Nop15 constructs (no-ENC, 1xENC, *i.e.*, WT, and 2xENC) to investigate the impact of the ENC size and the distance of the ENC to the RRM (no-linker) on protein stability at the physiological ionic strength (Fig. 2*B*). Protein stability was measured using fluorescence intensity ratio between tyrosine and tryptophan (FirbY-W) (35). Compared with the no-ENC construct (*ΔG* = 3.7 kcal·mol^−1^), the intramolecular interactions mediated by the Nop15 ENC increased protein stability by 0.7 kcal·mol^−1^ (Table 1). Moving the ENC closer to the RRM (no linker) or doubling the ENC length (2xENC) increased the protein stability to 4.9 and 5.7 kcal·mol^−1^, respectively. These increases in protein stability are expected, as moving the ENC closer or elongating the ENC increases the local concentration of the ENC and facilitates the intramolecular interactions. These unfolding results suggest the direct linkage between protein stability and the energetics of these intramolecular interactions.

**Table 1:** Protein stabilities of Nop15 constructs at 150 mM NaCl

Nop15 construct	*ΔG* (kcal·mol^−1^)	*ΔΔG* (kcal·mol^−1^)	*m*-Value (kcal·mol^−1^·M^−1^)	Denaturation midpoint (M)
WT	4.4 ± 0.2	0.7	1.38 ± 0.08	3.2 ± 0.1
no-ENC	3.7 ± 0.3	0	1.35 ± 0.11	2.7 ± 0.1
no-linker	4.9 ± 0.2	1.2	1.33 ± 0.05	3.7 ± 0.1
2xENC	5.7 ± 0.3	2.0	1.30 ± 0.07	4.4 ± 0.1

We hypothesize that interaction between the ENC and the RRM is electrostatic, and consequently salt sensitive. To test this hypothesis, we determined the protein stability at 500 mM NaCl (Fig. S2*A*). With the elevated salt concentration, the no-ENC construct has a similar stability to the ENC-bearing constructs or the no-linker construct (Table S2). These results suggest that the stability increases (*ΔΔG*) associated with intramolecular interactions are significantly reduced by ionic strength (Table S2). The stabilizing effect of ENCs can be partially mimicked by citrate, which also contains multiple carboxylic groups (Fig. S2*B*, Table S3).

To test whether the stabilizing effect of ENCs is generally applicable, we grafted an artificial ENC (EDEDEDEDED) to the second RRM domain of TDP-43 (TDP-43 RRM2) and to the RRM domain of SRSF3. These two proteins are orthogonal to Nop15 in that they (1) have no native ENC in their disordered regions and (2) only have a minimal basic site on the RNA-binding site (Fig. S2, *C* and *D*). As the two RRM proteins have no native tryptophan for FirbY-W assay, we used differential scanning calorimetry (DSC) to measure the protein stability. We found that introducing the artificial ENC increased the melting temperature of TDP-43 RRM2 by 2.4 degrees (Fig. 2*D*). A similar melting temperature increase was also observed for SRSF3 (Fig. S2*E*). These results suggest that the stabilizing effect of ENCs is generally applicable.

### ENCs suppress nonspecific RNA binding of Nop15

**Figure 3:**
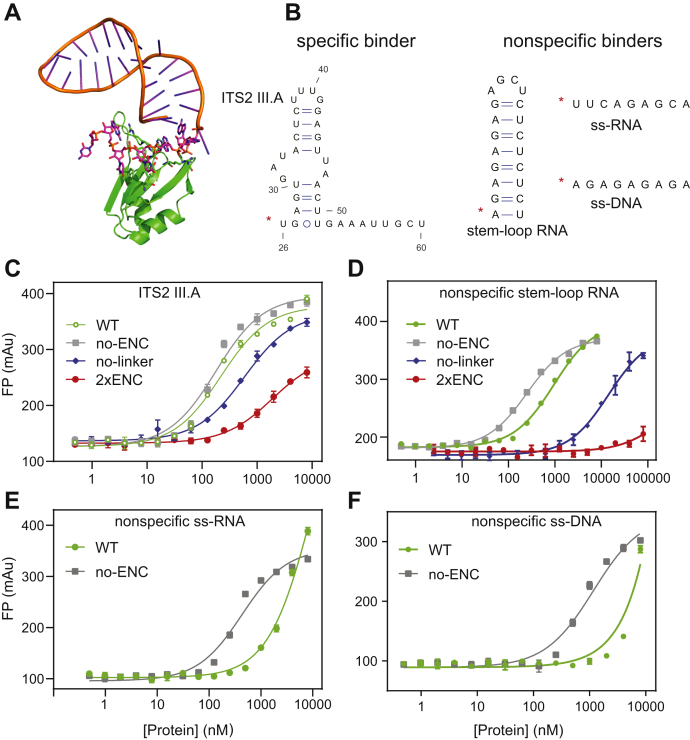
**The Nop15 ENC inhibits nonspecific RNA binding.***A*, cryo-EM structure of Nop15 and ITS2 III.A (region 26–60, PDB ID: 3JCT (36)). The ssRNA region and Nop15 residues involved in binding are shown as *orange* and *green sticks*, respectively. Other protein and RNA components are not shown for clarity. *B*, sequences and secondary structure of ITS2 III.A and nonspecific nucleic acids used in fluorescence polarization (FP) binding assays. The fluorescein is placed at the 5′ end of nucleotides as denoted by the *red asterisk*. *C*, FP binding assays of Nop15 constructs with specific RNA target ITS2 III.A. *D*, FP assays of nonspecific stem-loop RNA. *E*, FP assays of nonspecific ssRNA. *F*, FP assays of nonspecific ssDNA. The average curves were calculated from three individual measurements. ENCs, electronegative clusters.

Nop15 binds to ITS2 III.A RNA (nucleotides 26–60), which contains a stem-loop region and a 9-nucleotide single-stranded region (Fig. 3, *A* and *B*) (36). A previous structural and biochemical study has shown that both the stem loop and single-stranded regions contribute to binding of Nop15 (37). To test how ENCs affect RNA binding, we used fluorescence polarization (FP) assays to measure the RNA affinities of the four Nop15 constructs. Although the Nop15 ENC decreases specific RNA-binding affinity by only 1.2-fold relative to the no-ENC construct, bringing the ENC closer to the RRM (no linker) or doubling the length of the ENC (2xENC) decreases RNA binding by 3.4- and 10.8-fold, respectively (Fig. 3*C*, Table 2).

**Table 2:** RNA-binding affinities of Nop15 constructs measured by FP assays

RNA constructs	Nop15 construct's *K*_D_ (nM)				
	WT	No ENC	No linker		2xENC
Specific ITS2 RNA	215 ± 34 (1.2)^a^	173 ± 32 (1)		583 ± 90 (3.4)	1870 ± 270 (10.8)
Nonspecific stem-loop RNA	945 ± 65 (3.9)	244 ± 18 (1)		14,130 ± 1400 (57.9)	>80,000 (>327.9)
Nonspecific ssRNA	>8000 (>18.7)^b^	427 ± 98 (1)^b^		ND	ND
Nonspecific ssDNA	>8000 (>6.9)^b^	1162 ± 150 (1)^b^		ND	ND

ND denotes binding is too weak for detection.

a The values in the parenthesis are the relative *K*_D_ compared with the binding of the no-ENC construct. Mean *K*_D_ ± SEM from three technical replicates.

b The binding affinities were measured at 50 mM NaCl. Other measurements were carried out at 150 mM NaCl.

We continued to investigate how the ENC affects nonspecific RNA binding of Nop15. Here, we assumed that the nonspecific RNA binding is mainly mediated by the phosphate backbone. The specific binder of Nop15 consists of two key structural elements: a stem-loop region with eight Watson–Crick pairs and a 9-mer single-stranded region. Therefore, we selected RNA molecules that only resemble the backbone conformation of these two regions. The stem-loop region was mimicked by a stem-loop RNA of the same number of base pairs, but different nucleotides (Fig. 3*B*). The single-stranded 9-mer region was mimicked by an ssRNA or ssDNA of the same length, but different sequences (Fig. 3*B*). Without the ENC, the Nop15 RRM binds to the nonspecific stem-loop RNA (*K*_D_ = 244 nM), with a similar binding affinity to the specific RNA (*K*_D_ = 173 nM, Table 2). In contrast, the WT Nop15 and no-linker constructs bind to the specific RNA more than 4-fold and 57.9-fold tighter than it binds to the nonspecific stem-loop RNA, respectively (Fig. 3*D*, Table 2). Nonspecific RNA binding to the 2xENC constructs was beyond FP detection even at 80 μM protein, which reflects the lower limit of the *K*_D_ (Table 2). Nonspecific binding to ssRNA or ssDNA was detectable for the WT and no-ENC Nop15 constructs at 50 mM NaCl (Table 2). The binding affinities of WT Nop15 to nonspecific ssRNA or ssDNA could not be precisely determined because the ENC inhibition delays reaching of the FP plateaus (Fig. 3, *E* and *F*). However, these curves still provide lower boundaries for the *K*_D_ values. These results showed that Nop15 ENC also significantly inhibits nonspecific binding to single-stranded nucleic acids (Table 2).

### The Nop15 ENC interacts with the neighboring RRM through electropositive sites

**Figure 4:**
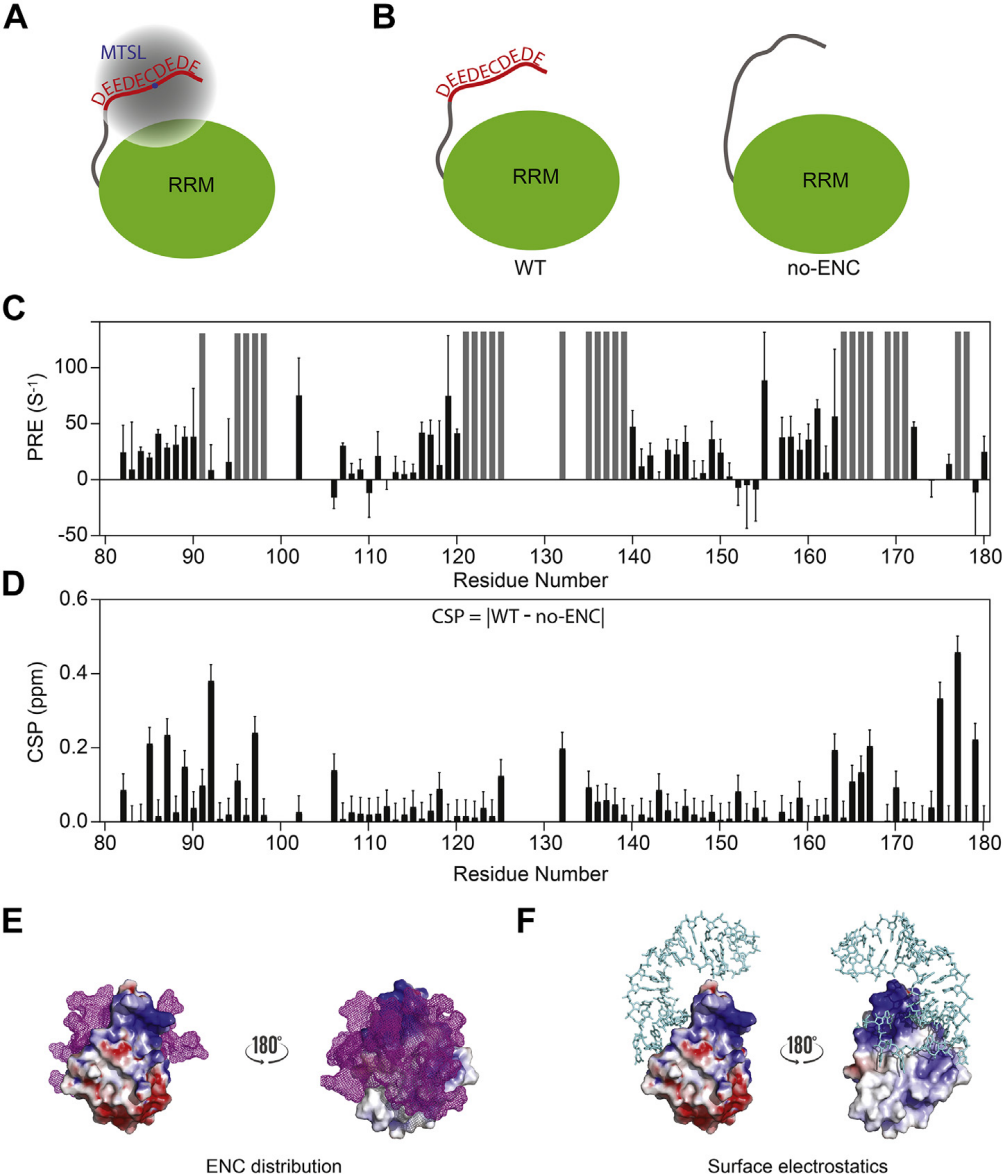
**The Nop15 ENC interacts with the RRM through electropositive sites.***A*, paramagnetic group MTSL was labeled in the middle of ENC (K45C). *B*, the protein constructs used to calculate chemical shift perturbation (CSP) by the Nop15 ENC. *C*, PRE values plotted along with Nop15 residues. *Gray bars* indicate the residues whose amide resonances disappear because of close proximity to MTSL. *D*, CSP calculated using |δ^1^H|+0.1∗| δ^15^N| for constructs shown *panel B*. The error was estimated by the resonance half width at the half height. *E*, distribution of the Nop15 ENC around the electrostatic surface of the Nop15 RRM with *red* and *blue* denoting the electronegative and electropositive surfaces, respectively. *Magenta mesh* represents the ten ENC conformers in the ensemble calculated by XPLOR-NIH. *F*, complex structure of Nop15 and ITS2 RNA (PDB ID: 3JCT). ITS2 III.A is shown as *cyan sticks*. The molecule orientations in *panels E* and *F* are identical. CSPs, chemical shift perturbations; ENCs, electronegative clusters; PRE, paramagnetic relaxation enhancement; RRM, RNA-recognition motif.

To track the dynamic behavior of the Nop15 ENC, we attached a nitroxide paramagnetic group (MTSL) in the middle of the ENC (Fig. 4*A*). The paramagnetic center enhances the ^1^H relaxation rate of NMR signals of the residues in its proximity, a phenomenon known as paramagnetic relaxation enhancement (PRE). PRE is powerful in detecting long-range (up to 25 Å) and transient interactions. The magnitude of PRE is reciprocally correlated to the distance from the paramagnetic center to the site of interest (38). In addition, we compared the chemical shifts of the WT Nop15 and the no-ENC mutant (Fig. 4*B*). As chemical shifts are sensitive to the local environment of nuclei, the intramolecular interaction of the ENC will perturb the microenvironment of the RRM and consequently cause chemical shift perturbations (CSPs). Therefore, CSP analysis is complementary to PRE in probing local information.

We collected heteronuclear single quantum coherence spectra for Nop15 in the MTSL-labeled (paramagnetic and diamagnetic) and MTSL-unlabeled states. The resonances of these three samples have identical peak positions, suggesting that ascorbate quenching and/or MTSL labeling does not change Nop15 structure (Fig. S3, *A* and *B*). The PRE values are plotted against the residue number (Fig. 4*C*). Some RRM residues undergo resonance disappearance (*gray bars* in Fig. 4*C*), suggesting these residues are within 12 Å to the ENC paramagnetic center (38). The CSP pattern resembles the PRE data but demonstrates more localized perturbation (Figs. 4*D* and S3*D*). To confirm that the intramolecular interactions are driven by the ENC, we compared the PRE difference (ΔPRE) between the WT and the no-ENC construct (PRE^WT^ – PRE^no-ENC^, Fig. S3*E*). The residues with resonance disappearance were assumed to have a PRE value of 100 s^−1^, which is typically one order of magnitude lower than the actual values (38). Positive ΔPRE values are mainly observed for the residues undergoing resonance disappearance, that is, the electropositive sites, suggesting the ENC enhances the interaction between the N-terminal region and the RRM. Negative ΔPRE values are observed for the residues that show moderate PRE values (<25 s^−1^) in Figure 4*C*, that is, the nonelectropositive surface on the Nop15 RRM. These negative ΔPRE values reflect the fact that without the ENC, the N-terminal disordered region has a higher probability to perturb nonelectropositive sites. Using distance restraints generated by PRE and CSP analysis, we used XPLOR-NIH to calculate the conformational ensemble for the Nop15 ENC (Fig. 4*E*). The ENC conformational ensemble can be found on the majority of the electropositive surface of the Nop15 RRM, including the RNA-binding site (Fig. 4*F*). Therefore, these PRE and CSP results suggest that the interaction between the ENC and the Nop15 RRM is through electrostatic interactions.

To verify the stability of conformers calculated by XPLOR-NIH and to dissect the energetic contributions of the intramolecular interactions, three representative conformers from the calculated ensemble were used as initial models for molecular dynamics (MD). 40 ns MD simulations were run followed by relaxing the system with explicit solvent molecules. The RMSD results are displayed in Figure S4. The dynamical movie of the three binding conformations is shown in Movies S1–S3. The RMSD values quickly increased in the first few nanoseconds and gradually drifted up in the rest of the simulations. The drift is partially due to the dynamic nature of the ENC/RRM interaction. The relative large drifts in conformations 1 and 2 are attributed to the ENC/RRM linker residues 51 to 89, which experience melting of two short helices during simulation (Movies S1 and S2). By contrast, the short helices preserved for conformation 3 explains the relatively small RMSD drift. Although the ENC fluctuated fast in their binding sites, no dissociation from the RRM was observed within the 40-ns simulation, indicating a stable ENC/RRM. The MD snapshots at 40 ns were selected as representative structures for the three possible binding patterns between the ENC and RRM (Fig. S4*C*). In particular, residues R132, K178, and K181 on the RNA-binding sites are the primary locations for the intramolecular interactions (36).

Note that compared with rigid docking analysis, the MD-equilibrated structures include structural relaxation and thermal motion at room temperature (RT), as well as solvent interactions. However, the three conformations of the ENC with the RRM can be observed within at least the tens of nanoseconds timescale, indicating their thermal stability. Meanwhile, we identified the residues contacting the ENC for the three representative conformations (Table S4). As expected, both charged and noncharged residues were found at the binding interfaces. Based on the MD production trajectories, we found that the average electrostatic interaction energies for the three binding conformations were -664, -594, and -399 kcal/mol, respectively; the average van der Waals interaction energies for the three binding conformations were -31, -38, and -42 kcal/mol, respectively (Table S4). Owing to the electrostatic interactions being at least an order of magnitude stronger than the van der Waals interactions, we concluded that the electrostatic interaction is the dominating driving force. These results are consistent with our finding that high ionic strength significantly decreases the stabilizing effect of the Nop15 ENC (Fig. S2*A*).

### An engineered ENC changes the landscape of RNA-binding specificity for SRSF3

**Figure 5:**
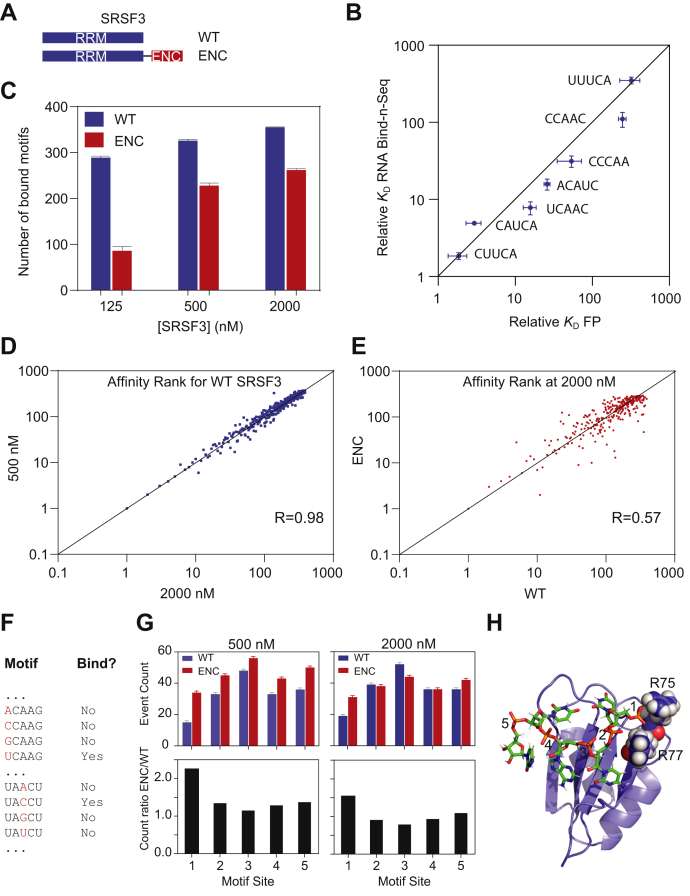
**An engineered ENC increases RNA-binding specificity of SRSF3.***A*, domain architecture of WT SRSF3 RRM and the mutant with an engineered ENC. *B*, correlation of relative dissociation constants (*K*_D_) measured by RNA Bind-n-Seq and fluorescence polarization. *C*, number of motifs pulled down by WT and ENC SRSF3 at different protein concentrations. *D*, rank correlation of RNA motifs bound to SRSF3 at 500 nM and 2000 nM. *E*, rank correlation of RNA motifs bound to WT and ENC SRSF3 at 2000 nM. *F*, grouping motifs for analysis of site specificity. In each group, only the sites to be examined are different. Two discriminating events are shown as examples. *G*, discriminating events identified at 500 nM and 2000 nM SRSF3 (*top*) and event count ratio of ENC over the WT SRSF3 (*bottom*). *H*, structure of SRSF3–RNA complex (PDB ID: 2I2Y). SRSF3 RRM is shown in *blue* cartoons, and RNA is shown as *green sticks*. Sites 1 to 5 are labeled for RNA (5′-UCAUC-3′). The arginine residues involved in phosphate backbone interactions are shown as *spheres*. 5′ U is simulated on the basis of the original 5′-CAUC-3′ sequence to help visualization of phosphate backbone at site 1. ENCs, electronegative clusters; RRM, RNA-recognition motif; SRSF3, Ser/Arg-rich splicing factor 3.

The RNA recognition of Nop15 is *via* both the overall shape and sequence of the stem-loop RNA (37). It is common that many RBPs recognize single-stranded RNA ligands and can potentially bind to different RNA motifs. Although we have shown that the Nop15 ENC inhibits nonspecific RNA binding, the question remains as to how an ENC affects sequence specificity of RNA binding. With regard to this question, Nop15 is not the optimal model, as RNA secondary structure also plays a role in binding. To answer this question, we grafted an engineered ENC to the C-terminal end of SRSF3 (Fig. 5*A*). The SRSF3 RRM binds to ssRNA. In addition, SRSF3’s electropositive surface is mainly confined to the RNA-binding site, eliminating the problem of nonspecific RNA binding by non–RNA-binding sites (Fig. S2*D*). Previous studies have shown that the SRSF3 RRM binds to 5-mer pyrimidine-rich sequences using SELEX and iCLIP (39, 40, 41). These methods are powerful to identify the strongest RNA motifs, while weaker ones cannot be captured. To study the effect of the engineered ENC on RNA-binding specificity, the relative binding affinities of all RNA motifs that bind to SRSF3 have to be determined. To this end, we carried out RNA Bind-n-Seq on WT and ENC-mutant SRSF3 at different protein concentrations (42). The ENC mutant pulls down less RNA compared with the WT protein (Fig. S5*A*). This difference reflects the fact that the ENC mutant has a lower RNA-binding affinity. As the background RNA binding by the resin accounts for about 50% of the pulled-down RNA for the ENC mutant at 125 nM, the data from this protein concentration were excluded in determination of relative binding affinities (Fig. S5*A*).

As detailed in the Experimental procedures section, we confirmed that 5-nucleotide-long motifs are necessary and sufficient for specific SRSF3 binding. The top 2% of motifs identified by our experiments cover the ones revealed by previous iCLIP and SELEX studies (Table 3) (39, 40, 41). To further crosscheck the validity of RNA Bind-n-Seq, we chose 5-mer motifs of different relative binding affinities and measured their binding affinities using FP. The relative binding affinities determined by RNA Bind-n-Seq match the ones determined by FP assays (Figs. 5*B* and S5*B*). All these results confirm the validity and robustness of our approach.

**Table 3:** Top 20 motifs identified by RNA Bind-n-Seq

Motif	*K*_D_^Rel^	Rank	iCLIP^a^	SELEX^b^
CUCCC	1	1		
CUACA	1.1	2		
CAACA	1.8	3		
CUUCA	1.9	4	x	x
UCCCC	2.8	5		
CAUCA	4.9	6	x	x
ACUCC	5.0	7		
CCCCC	5.0	8		
UCUAC	5.4	9		x
CACCA	7.4	10		
CACCC	7.5	11		
UCAAC	7.8	12	x	x
UCCCA	8.4	13		
ACAAC	8.7	14		x
UCUUC	8.8	15		x
CUCCU	9.6	16		
ACUAC	10.4	17		x
CAUCU	14.6	18		x
ACAUC	15.8	19		x
CCCCA	15.9	20		

a Motifs identified in reference (41).

b Motifs identified in reference (39).

We further analyzed the number of motif types that SRSF3 binds at different protein concentrations (Fig. 5*C*). The number of motif types pulled down by SRSF3 increases along with protein concentration as predicted, and WT SRSF3 pulled down more motifs than the ENC mutant (Fig. 5*C*). It is noteworthy that the same amount of cDNA for each library was used for deep sequencing. Therefore, the decrease in the number of motif types pulled down by SRSF3 indicates an increased RNA-binding specificity by the ENC. Based on the relative *K*_D_ values, we further determined the affinity rank of the RNA motifs. The affinity rank reflects the relative affinities of various RNA motifs. As shown in Figure S5*C*, the ranking patterns for the WT and ENC-mutant SRSF3 show that the motifs containing C, A, and U are preferred more than those with G. However, the rank patterns show dissimilarities for weaker binding motifs. To provide a more straightforward visualization, we plotted the affinity ranks for the RNA motifs bound to WT SRSF3 at 500 and 2000 nM. This plot demonstrates a high correlation value (R = 0.98), which is expected and indicates the robustness of the analysis (Fig. 5*D*). However, the affinity rank correlation between WT and ENC-mutant SRSF3 is significantly lower (R = 0.57, Fig. 5*E*). These dissimilarities in the motif ranking suggest that the engineered ENC inhibits different motifs to different extents, instead of weakening all motifs to the same extent.

We further analyzed the contribution of each of the five nucleotide sites to sequence specificity. To this end, the 1024 RNA motifs were grouped in such a way that each group only differs in one site (Fig. 5*F*). The groups that contain one and only one motif that binds to SRSF3 were identified (Table S5). For example, in group UAXCU, only motif UACCU binds with SRSF3, indicating site 3 requires a cytidine for binding as any mutation in this site abolishes binding. We defined identification of such groups as discriminating events. The occurrence of discriminating events reflects how “discerning” the site is for binding. More discriminating events were identified for the ENC mutant than WT SRSF3 at both concentrations (Fig. 5*G*, top), which is consistent with our finding that the grafted ENC increases RNA-binding specificity for SRSF3 (Fig. 5*C*). Our analysis also shows that site 3 has more discriminating events than others, which agrees with the previous study that site 3 has the highest conservation (41).

By calculating the ratio of discriminating events for the ENC mutant and WT SRSF3, we compared the relative change in discriminating events by introduction of the ENC. For both protein concentrations, site 1 shows the highest ratio, suggesting that specificity is increased more for this site by the engineered ENC (Fig. 5*G*, bottom). Coincidently, structural analysis shows that site 1 is adjacent to two basic residues, R75 and R77, which constitute the electropositive surface for RNA binding (Fig. 5*H*). This finding indicates that the site involved in electrostatic interactions is more susceptible to ENC regulation.

## Discussion

Our study revealed an unexpected finding that ENCs are the most abundant repetitive sequences in RBPs’ disordered regions. Considering the fact that numerous phosphorylation sites have yet to be identified, and that some structured RNA-binding domains have embedded ENCs (43), the occurrence of ENCs is actually higher than reported here. The high occurrence of ENCs is unlikely to result from stochastic processes as shown by the low *p*-values. In addition, ENCs are more often found immediately adjacent to, rather than within, RNA-binding domains. It seems that evolution selected this location to avoid destabilizing protein by the loop-closure entropy (44).

Concomitant with unawareness of ENCs’ abundance is our poor understanding of their regulatory functions, which have only been investigated by scattered studies (25, 26, 27, 29). Although some ENCs may perform regulatory roles by recruiting binding partners, this is unlikely to be a general mechanism as most ENCs differ only in length. If recruiting binding partners was the general mechanism by which ENCs function, promiscuity would be a problem. Indeed, the Nop15 ENC is invisible in the cryo-EM structure of ribosomal pre-60S complex (PDB ID: 3JCT (36)), suggesting that the ENC is unlikely to function by forming stable contacts with other protein or RNA components.

We proposed that ENCs regulate neighboring RNA-binding domains for their binding affinity and specificity through intramolecular interactions. Here, we provided a way to measure the dynamic intramolecular interaction between ENCs and RNA-binding domains through its coupling with protein stability. We also found that ENCs’ stabilizing effect is generally applicable.

### The role of ENCs in modulating RNA-binding specificity

RBPs need to balance between binding affinity and specificity. This balance is realized by a tradeoff between electrostatic interactions and nonelectrostatic ones, such as H-bonds and stacking interactions. Most RBPs use electropositive surfaces to enhance RNA binding. However, overuse of electrostatic interactions also increases nonspecific binding, as seen with the Nop15 RRM.

Nop15’s function is to stabilize a transiently existing stem-loop RNA structure. Considering the omnipresence of stem-loop structures in ribosome biogenesis (30, 31), without the ENC, nonspecific binding could hinder Nop15 from forming the specific complex. Part of nonspecific binding may stem from the electropositive non–RNA-binding surface (1160 Å^2^), which accounts for roughly half of the total electropositive surface (2392 Å^2^) based on solvent-accessible surface area analysis (45). As shown by our MD simulations, the native ENC can roughly occupy the RNA-binding site (Fig. S4). Therefore, the ENC may mainly interact with the electropositive non–RNA-binding surface and not significantly compete with the RNA ligand in the bound complex. This may explain the negligible inhibition of the native ENC on specific RNA binding. Doubling the native ENC (2xENC) increases its inhibitory effect on the specific RNA ligand by 9-fold. Considering the fact that the elongated ENC matches the size of the entire electropositive surface of Nop15, 2xENC likely inhibits both the RNA-binding site and the non–RNA-binding site. Compared with the native ENC, inhibition of 2xENC to nonspecific stem-loop RNA binding was increased by larger than 84-fold (Table 2).

It is intuitive to expect that ENCs inhibit RNA binding. However, our results on SRSF3 revealed that the inhibitory effect is discriminating instead of weakening all binders to the same extent. The site involved in electrostatic interactions between RNA phosphate backbone and basic protein residues is more susceptible to the inhibition. Our finding that the ENC can reshape the landscape of RNA-binding specificity for SRSF3 has implications to the RBPs with the phosphorylatable ENCs. For these RBPs, phosphorylation of ENCs could adjust not only the RNA binding affinity but also specificity.

**Figure 6:**
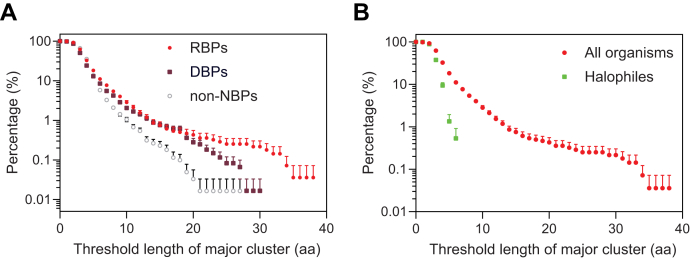
**ENCs are overrepresented in DNA-binding proteins and underrepresented in halophilic proteins.***A*, occurrence of ENCs in RBPs, DNA-binding proteins (DBPs), and non–nucleic acid–binding proteins (non-NBPs). *B*, occurrence of ENCs in halophiles compared with all organisms. ENCs, electronegative clusters; RBPs, RNA-binding proteins.

Because DBPs also need to deal with nonspecific binding, we predict that ENCs are also enriched in DBPs. Therefore, we compared the occurrence of ENCs in RBPs, DBPs, and non-NBPs. Consistent with our prediction, we found that the occurrence of ENCs in RBPs and DBPs is higher than that in non-NBPs (Figs. 6*A* and S6*A*). This finding is consistent with a recent study by Krois and Wright that the acidic N-terminal disordered region of p53 inhibits nonspecific DNA binding (46).

Nonspecific RNA binding is less problematic for halophiles because halophilic proteins perform their functions at salt concentrations in the range of molars (47). Nonspecific RNA binding should be largely prevented by high salt. In addition, a high concentration of salt prevents ENCs from regulating RNA-binding domains by abolishing intramolecular interactions (Fig. S2*A*). The results outlined above suggest that ENCs are less essential in halophilic RBPs and would therefore be less abundant. To test this, we compared the ENC occurrence and *p*-values of halophiles with those of other organisms (Figs. 6*B* and S6*B*). Strikingly, we found that ENCs in halophiles are shorter and their occurrence is significantly lower than in other organisms. This is in spite of the fact that the composition of acidic residues is much higher in halophiles than it is in other organisms (48, 49). Therefore, the high occurrence of ENCs in RBPs or the low occurrence of ENCs in halophiles is not attributed to the amino acid composition. These results suggest that ENCs do not occur at random and may be selected by evolution for their functions.

## Experimental procedures

### Bioinformatic analysis of repetitive sequences in RBPs

Protein sequences, domain annotations (domain name and starting and ending residues), and reported phosphorylation sites of Ser, Thr, and Tyr were obtained from UniProt (https://www.uniprot.org/) (32). Domains in UniProt are defined by PROSITE, Pfam, and SMART (50, 51, 52). In total, 2783 RBPs, 6057 DBPs, 6087 non-NBPs, and 373 halophilic proteins that have domain annotations were analyzed by in-house python scripts. The regions that are not annotated as domains were assumed to be disordered. ENCs were defined as the sequences that contain consecutive electronegative residues, that is, Glu, Asp, and/or phosphorylated Ser, Thr, or Tyr sites. Using the same criterion, poly-K/R, poly-G, poly-N/Q, poly-F/W/Y, poly-I/L/V, poly-S/T, and other homopolymers (poly-C, poly-H, poly-M, poly-A, poly-P) were also analyzed for RBPs, DBPs, and non-NBPs. Considering the fact that a protein can contain multiple repetitive sequences of different lengths (in the number of amino acids), only the longest one(s) (major clusters) were counted. The error of the occurrence is estimated by $\frac{1}{\sqrt{n}}$ , where *n* is the number proteins whose major clusters pass a given threshold length. The mole percentage of an amino acid is calculated as the count of the specific amino acid over the total amino acid count in the disordered protein regions of all proteins analyzed. Monte Carlo simulations were used to determine the probability by which a consecutive major cluster occurs at random in disordered regions of RBPs. Given the amino acid composition of a protein, 100,000 sequences for the disordered regions were generated at random. The occurrence of the sequences harboring clusters equal to or longer than the threshold value was counted as the *p*-value for each protein. ENC and poly-N/Q longer than 11 residues, poly-G longer than ten residues, poly-A longer than eight residues, poly-S/T longer than 11 residues, and poly-P longer than ten residues were not found among the 100,000 simulations. Therefore, the *p*-values of these clusters are lower than 0.001%.

### Protein expression and purification

#### Nop15

The yeast Nop15 (UniProt accession number P53927, residues 40–191) gene was amplified from *Saccharomyces cerevisiae* genomic DNA using PCR and cloned into pSMT3 (provided by Christopher Lima, Memorial Sloan Kettering Cancer Center, New York, NY). Nop15 mutants, such as K45C, no linker (residues 60–76 deleted), 2xENC (residues 39–49 duplicated), and no-ENC (residues 39–49 replaced by SGGSSGKSGSG), were created by mutagenesis PCR. Nop15 constructs were expressed at 22 °C overnight in *Escherichia coli* strain BL21-CodonPlus (DE3) using 0.5 mM IPTG, which was added when the absorbance at 600 nm reached 0.6 AU at 37 °C (0.8 AU for ^13^C-,^15^N-, and ^2^H-labeled samples). Once pelleted, the cells were resuspended in 25 mM Tris HCl, pH 8.5, 1 M NaCl, 25 mM imidazole, 1 mM PMSF, 0.5 mg/ml lysozyme, 1 protease inhibitor tablet (Thermo Fisher Scientific), and 0.2 mM TCEP and subjected to three freeze-thaw cycles. The cells were lysed by sonication and centrifuged at 23,710 relative centrifugal force at 4 °C for 45 min to remove cell debris. The supernatant was applied to 5 ml of Ni Sepharose excel resin (GE Healthcare) and washed with 200-ml loading buffer (25 mM Tris HCl, pH 8.5, 1 M NaCl, 25 mM imidazole, and 0.2 mM TCEP) followed by 10 ml of 25 mM Tris HCl, pH 8.5, and 0.2 mM TCEP. The protein was eluted using 25 mM Tris HCl, pH 8.5, 500 mM NaCl, 500 mM imidazole, and 0.2 mM TCEP. The N-terminal SUMO tag was cleaved using 0.1 mg Ulp1 and incubated for 3 h at 25 °C or overnight at 4 °C. The sample was diluted 2-fold using 20 mM Tris HCl, pH 7.5, and 1 mM TCEP and loaded onto a 5-mL HiTrap Heparin Column (GE Healthcare). The sample was eluted with a gradient from 0 to 2 M NaCl in 20 mM Tris HCl, pH 7.5, and 0.2 mM TCEP. Nop15 was further purified using a HiLoad 16/60 Superdex 75 column (GE Healthcare) equilibrated in 21 mM MES, pH 5.5, 105 mM NaCl, 420 mM Arg/Glu, and 0.3 mM TCEP. The purities of these proteins were higher than 95% based on SDS-PAGE.

### RRM2 of TDP-43

The human TDP-43 RRM2 (UniProt accession number Q13148, residue 190–261) and TDP-43 RRM2 with an artificial C-terminal ENC (EDEDEDEDED) were cloned, expressed, lysed, and subjected to Ni Sepharose purification in the same way as Nop15. The eluted sample from Ni-Sepharose was diluted 5-fold in 20 mM Tris HCl, pH 7.5, and 1 mM TCEP and loaded onto a 5-mL HiTrap Q Column (GE Healthcare). The protein was eluted with a gradient from 0 to 2 M NaCl in 20 mM Tris HCl, pH 7.5, and 0.2 mM TCEP. The RRM2 fractions were concentrated to 5 ml. The concentrated sample was loaded to a HiLoad 16/60 Superdex 75 column (GE Healthcare) equilibrated in 20 mM Hepes, pH 7.5, 150 mM NaCl, and 0.3 mM TCEP. The purities of these proteins were higher than 95% based on SDS-PAGE.

### SRSF3

The human SRSF3 (UniProt accession number P84103, residue 1–84) was cloned, expressed, lysed, and loaded to Ni Sepharose resin in the same way as Nop15. After washing with 200 ml of 20 mM Tris HCl, pH 8.0, 4 M NaCl, and 0.1 mM TCEP, the resin was resuspended in 10 ml of 20 mM Tris HCl, pH 7.5, 2 M NaCl, 25 mM imidazole, 0.2 mM TCEP, and 0.01 mg/ml Ulp1 for overnight on-column cleavage at 4 °C. The cleaved sample was concentrated to 5 ml before loading to a HiLoad 16/60 Superdex 75 column equilibrated with 20 mM Hepes, pH 7.5, 150 mM NaCl, and 0.2 mM TCEP. The C-terminal ENC mutant (GSGSEDEDEDEDED) was prepared by mutagenesis PCR and purified the same way as the WT protein. Streptavidin-binding peptide (RGGHVVEGLAGELEQLRARLEHHPQG) was inserted between the SUMO tag and SRSF3 using mutagenesis PCR. The SBP-tagged SRSF3 was purified using the same protocol as the WT protein. The purities of these proteins were >95% based on SDS-PAGE.

### Nop15 unfolding analysis by FirbY-W

Trp fluorescence data were collected using a Varian Cary Eclipse fluorometer at 25 °C with a 5-mm cuvette to measure protein unfolding by FirbY-W (fluorescence intensity ratio between tyrosine and tryptophan) (35). Protein samples (600 μl, 10 μM) were equilibrated in 20 mM Tris HCl, pH 7.5, 0.1 mM TCEP, 100 or 500 mM NaCl, and various urea concentrations ranging from 0 M to 7 M. The samples were centrifuged at 10,000 relative centrifugal force for 10 min at 4 °C before data collection. The excitation wavelength was set to 275 nm, and spectra from 280 nm to 400 nm were collected with 5-nm excitation and emission slits. The fluorescence intensity at 302 nm was used in unfolding analysis. After subtraction of background fluorescence, the tyrosine and tryptophan fluorescence were deconvoluted as described in the previous study (35). Using the deconvoluted fluorescence emission maximums for the Tyr and Trp spectra, the FirbY-W values were calculated for each urea concentration and fitted to the following equation:

$$\text{FirbY}-\text{W}=\frac{De^{\left(\frac{mX-\Delta G}{RT}\right)}+N}{e^{\left(\frac{mX-\Delta G}{RT}\right)}+1}$$

Where *ΔG* is the unfolding energy of proteins; *R* is the gas constant; *T* is the temperature (295 K); *N* and *D* are the FirbY-W values at the native and denatured states, respectively; *X* is the urea concentration in molar, and *m* is the *m*-value for urea. The errors were estimated from curve fitting.

### FP assays

FP assays were carried out using 10 nM 5′ fluorescein-labeled RNA mixed with Nop15 constructs at concentrations ranging from 8000 nM to 0.488 nM by 2-fold serial dilutions in 20 mM Tris HCl, pH 7.5, 0.02% Tween 20, and 150 or 50 mM NaCl. The sequences of RNA and DNA (product of Dharmacon) were as follows: ITS2 III.A 26-60 (UGAGUGAUACUCUUUGGAGUUAACUUGAAAUUGCU), nonspecific RNA (UUCAGAGCA), and nonspecific ssDNA (AGAGAGAGA), and nonspecific stem-loop RNA (AGAGAGAGAGUCUCUCUCUC). The 5-mer RNA oligos with 5′ fluorescein label (CUUCA, CAUCA, UCAAC, ACAUC, CCCAA, CCAAC, and UUUCA) for SRSF3 FP binding assays were purchased from IDT and used without further purification. The binding assays were performed in a buffer containing 10 mM MES, pH 5.5, 50 mM Arg/Glu, 0.1 mM TCEP, and 0.02% Tween 20. The 100-μl samples were mixed in black flat-bottom 96-well plates (Costar) by shaking at 100 RPM for 5 min, followed by incubation at 37 °C for 30 min, and incubation at 25 °C for 20 min. All binding assays were repeated three times to estimate the error.

The FP data were gathered at RT using a BioTek Synergy 2 plate reader with an excitation wavelength of 485 nm and an emission wavelength of 520 nm. The binding affinities were determined using nonlinear regression for one-site interaction using GraphPad Prism 7. The FP anisotropy (*F*_*p*_) was fitted using the quadratic equation below, where the fitting parameters *F*_*min*_, *F*_*max*_, and *K*_*D*_ are the FP anisotropy baseline, plateau, and dissociation constant, respectively. [*P*_*T*_] is the total protein concentration, and [*L*_*T*_] is the total RNA concentration (10 nM). Errors in the dissociation constants were calculated based on three independent measurements.

$$F_{p}=F_{min}+\left(F_{max}-F_{min}\right)\left\{\frac{\left[\left(\left[P_{T}\right]+\left[L_{T}\right]+K_{D}\right)-{\left\{{\left(\left[P_{T}\right]+\left[L_{T}\right]+K_{D}\right)}^{2}-4\left[P_{T}\right]\left[L_{T}\right]\right\}}^{0.5}\right]}{2\left[L_{T}\right]}\right\}$$

### DSC

DSC experiments were performed on a MicroCal MC-II differential scanning calorimeter (GE Healthcare) at a protein concentration of 1 mg/ml for the TDP-43 RRM2 and 0.5 mg/ml for the SRSF3 RRM in 20 mM Hepes, pH 7.5, 150 mM NaCl, and 0.3 mM TCEP. The buffer without proteins served as the control. DSC data were recorded from 40 to 110 °C at a scanning rate of 30 °C/h. The experiments were repeated on protein samples that were purified in three individual preparations to estimate the errors of the melting temperature. The melting temperature was calculated using the Origin software package (MicroCal).

### NMR assignment experiments

The Nop15 construct (residue 81–180) was prepared as described above except that the *E. coli* cells were grown in M9 media containing ^15^N, ^13^C, and ^2^H isotopes. The protein (∼635 μM) was purified as described above and exchanged into 20 mM MES, pH 5.5, 400 mM arginine/glutamic acid, 100 mM NaCl, and 5% D_2_O for NMR measurements. Triple resonance assignment experiments HNCA, HNCACB, HN(CO)CA, CBCA(CO)NH, and HNCO were collected at 25 °C on a Bruker Avance III-HD 850-MHz spectrometer installed with a cryo-probe. The NMR data were processed using NMRPipe (53), and the assignment was performed using NMRViewJ (54). The backbone resonances were assigned except residues 99 to 101, 103 to 104 and 125 to 131, and 133 to 134. The assignment has been submitted to Biological Magnetic Resonance Data Bank (ID: 50271).

### PRE

^1^H PRE data were gathered at 25 °C on a Bruker AVANCE III-HD 600-MHz spectrometer installed with a cryo-probe. The protein construct Nop15 40-191 K45C no linker was prepared as described above except that the *E. coli* cells were grown in M9 media containing ^15^NH_4_Cl. Immediately before paramagnetic labeling with MTSL, TCEP was removed by loading the sample onto a HiPrep 26/10 desalting column (GE) equilibrated with 20 mM Tris HCl, pH 7.5, 100 mM NaCl, and 400 mM arginine/glutamate. The protein was diluted to 40 μM and mixed with 200 μM MTSL for overnight reaction at 4 °C. Unreacted MTSL was removed by loading the sample onto a HiPrep 26/10 desalting column (GE) equilibrated in 20 mM MES, pH 6.0, 400 mM arginine/glutamic acid, and 5% D_2_O. The PRE measurements were carried out using a pulse sequence developed by Junji Iwahara (38). A total of 64 scans were accumulated, and the relaxation time interval was set to 8 ms. Diamagnetic data were collected with the above sample quenched using 2 mM ascorbic acid. The NMR data were processed using NMRPipe (53) and analyzed using NMRViewJ (54). The errors were estimated from PRE measurements of two independent samples.

### Nop15 ensemble structure calculations

The structure of Nop15 (residue 40–184 Δ60–76) was calculated with XPLOR-NIH using a restrained rigid body–simulated annealing protocol refined against the PRE and CSP data (55, 56). The ENC and linker region (residues 40–92) was allowed all torsion angle degrees of freedom, while the backbone of the RRM domain (residues 92–184) was held rigid, and only side-chain atoms allowed torsion angle degrees of freedom. The MTSL paramagnetic probe was represented with three conformers to account for linker flexibility. An ensemble representation of Nop15 conformers was used to fit the PRE and CSP data (57). Quantitative agreement between the observed (Γ_2_^obs^) and calculated (Γ_2_^calc^) PRE relaxation rates was measured using Q-factor, calculated by using the following equation.

$$Q=\sqrt{\frac{\sum_{i}{\left\{\Gamma_{2}^{obs}\left(i\right)-\Gamma_{2}^{calc}\left(i\right)\right\}}^{2}}{\sum_{i}\Gamma_{2}^{obs}{\left(i\right)}^{2}}}$$

where *i* is the residue number. CSP values were calculated by comparing the chemical shifts of no-ENC and WT Nop15 using the formula |δ^1^H|+0.1∗| δ^15^N|. CSP values >0.1 ppm were used as ambiguous distance restraints. The bleached amide protons were restrained within 15 Å to the MTSL tag. In the structure calculation, the degrees of freedom were initially randomized and gradient-minimization was performed, followed by a standard simulated annealing protocol. 100 ensembles were calculated with ensemble sizes ranging from 9 to 15. The Q-factors for ensembles with 9, 10, 11, and 15 conformers are 0.32, 0.15, 0.32, and 0.20, respectively. Therefore, the best ensemble contains ten conformers and was further analyzed. The scripts and parameters used throughout this structure calculation can be obtained upon request.

### MD simulations

Three conformers calculated from XPLOR-NIH were used as starting model for MD simulations. NVT ensemble MD simulations were carried out using NAMD2.9, starting with the three initial structures obtained from the molecular docking studies solvated with explicit TIP3P water molecules (58, 59). The simulation temperature was set at 25 °C with a damping coefficient γ = 5 ps^−1^. The cutoff distance of nonbonded interactions was set to 12.0 Å, and the corresponding switching and pair list distances were set to 10 Å and 14.0 Å, respectively. The AMBER ff12SB force field parameters were used for the protein (60). Full electrostatics was used using the particle-mesh Ewald method with a 1 Å grid width (61). In the simulations, the nonbonded interactions were calculated using a group-based cutoff with a switching function and were updated every ten time steps. Covalent bonds involving hydrogens were held rigid using the SHAKE algorithm, allowing a 2-fs time step (62). Each trajectory was equilibrated for 20 ns with an additional 20-ns production period.

### RNA pull-down and next-generation sequencing sample preparation

The T7 template (Table S1) annealed to the T7 promoter serves as a template for *in vitro* transcription of RNA. A 40-nt randomized region was introduced into the template to produce corresponding RNA. The *in vitro* transcription samples were incubated at 37 °C overnight in 100 mM Tris HCl, pH 8.5, 20 mM MgCl_2_, 1 mM TCEP, 2 mM spermidine, 3% PEG 8000, 0.01% (v/v) Triton X-100, 4 mM nucleotide triphosphates, 2 units of inorganic pyro-phosphatase, 0.2 units of RNAse inhibitor, 0.6 μM dsDNA template, and 0.06 mg/ml T7 RNA polymerase. The RNA sample was purified by 6% polyacrylamide gel (30 cm x 40 cm x1.6 mm) in the presence of 8 M urea and 1x TBE. The gel containing target RNA was eluted by electrophoresis in 1x TBE.

SRSF3 and its ENC mutant were incubated with 2 μM RNA at 25 °C for 30 min in 10 mM MES, pH 6.0, 50 mM Arg/Glu, 1 mM TCEP, 150 mM NaCl, 0.01% Tween 20, 0.4 units of RNAse inhibitor, and 0.05 mg/ml BSA. The protein–RNA complex was incubated for 30 min with 75 μl pre-equilibrated magnetic Dynabeads (Thermo Fisher) on a rotator at 25 °C. The pulled-down complex was washed with 0.5 ml of 10 mM MES, pH 6.0, 50 mM Arg/Glu, 1 mM TCEP, 150 mM NaCl, 0.01% Tween 20, and 0.5 mM EDTA for 1 min. The bound RNA was eluted from the beads by incubating at 70 °C for 10 min in 100 μl of 10 mM Tris HCl, pH 6.8, 1 mM EDTA, and 1% SDS. The eluted RNA was extracted by phenol and chloroform and subjected to overnight precipitation at -20 °C after adding 10 μl of 3 M sodium acetate (pH 5.2) and 300 μl of ice-cold 100% ethanol. The RNA sample was pelleted and dried before dissolving in 20 μl of 10 mM Tris HCl, pH 6.8, and 1 mM EDTA. The WT SRSF3 and ENC-mutant were prepared at three concentrations (2000 nM, 500 nM, and 125 nM). A negative control without protein was prepared following the same procedure to assess nonspecific RNA binding of the resin. The RNA quality and quantity were analyzed by an Agilent 2100 Bioanalyzer. The error of the Bioanalyzer analysis was estimated by the baseline of the sample lanes.

The RNA was reverse-transcribed into cDNA following the user manual of SuperScript IV Reverse Transcriptase (Thermo Fisher). Briefly, 5 μl of RNA template, 1 μl of 2 μM reverse transcriptase primer, 1 μl of 10 mM dNTP, and 6 μl water were mixed and annealed at 65 °C for 5 min before adding 4 μl of 5 x SSIV buffer, 1 μl of 100 mM DTT, 1 μl of RNAseout, and 1 μl of SuperScript IV. About 0.5 μl of 10 μM input RNA was reverse-transcribed into cDNA using the same procedure as a reference. The samples were incubated at 55 °C for 1 h. The 1 μl reverse-transcribed samples were mixed with 10.5-μl water, 0.5 μl of RP1 primer, 0.5 μl of corresponding index primers, and 12.5 μl of PrimeSTAR HS DNA polymerase premix for PCR amplification. The number of cycles was 12 for the negative control and 125 nM ENC samples, 10 for 2000 nM and 500 nM ENC samples, eight for input RNA and 125 nM WT SRSF3, and 6 for 500 nM and 2000 nM WT SRSF3. The PCR samples were purified by 5% polyacrylamide gel prepared in 1x TBE and visualized by SYBR Gold nucleic acid gel stain. The gel slices containing the target DNA (∼160 nt) were dialyzed in 400-uL water and quantified by an Agilent 2100 Bioanalyzer. The eight libraries were mixed in equal amounts and concentrated to a total concentration of 20 nM for Illumina HiSeq 2x 150 bp sequencing by GENEWIZ. A 30% PhiX was spiked in for quality control, and in total, 330,000,000 reads were sequenced. The sequencing data have been deposited in SRA (ID: SUB8809326).

### RNA Bind-n-Seq analysis

Analysis of the NGS data was similar to the method previously reported (42) with some modifications detailed below. The reads containing undetermined nucleotides (‘N’) amid the sequences or sequences shorter than 37 nucleotides were excluded from the analysis. Around 94% of reads passed the filtering standards.

### Determination of the minimal motif length necessary and sufficient for specific binding

The occurrence frequency of motif *i* ($f_{i\;}$) is defined as the ratio of the motif occurrence over the total occurrence of all possible motifs. The enrichment value (R-value) is defined as the frequency of the motif in the sample library ($f_{i,\;sample}$) over that in the input library ($f_{i,\;input}$), that is, $R=\frac{f_{i,\;sample}}{f_{i,\;input}}$. The magnitude of R value is positively correlated with binding affinity.

To determine the minimal motif length necessary and sufficient for specific binding, we compared the enrichment values (R-values) for the optimal *k*-mer motifs when *k* is 4, 5, and 6. The R-values were calculated as the ratio of the frequency of each *k*-mer in the selected library to the frequency in the input RNA library. When *k* is 4, 5, and 6, the optimal *k*-mer is CUCC, CUCCC, and ACUCCC with an R-value of 1.78, 2.44, and 2.68, respectively. The low R-value for the optimal 4-mer motif suggests that 4-mer is not long enough for specific binding. The optimal 6-mer ACUCCC contains the optimal 5-mer motif. Permutation of the first site to the sixth site (ACUCCC to CUCCCA) yields a similar R-value of 2.65, suggesting that SRSF3 does not discriminate the nucleotide outside of the CUCCC core. In contrast, permutation of CUCCC to CCUCC decreases the R-value from 2.44 to 1.75. Therefore, we confirmed that the 5-mer motif is the minimal length necessary and sufficient for specific binding.

### Determination of the enrichment values and relative binding affinities

We assume that SRSF3 binds to the strongest 5-mer motif in a given read with length of *N* among the *N-k*+1 possible *k*-mer motifs (*k* = 5 in this case). This assumption is valid as the input protein concentration is smaller than or equal to the total RNA concentration. The problem is that binding of the strongest 5-mer motif will also pull down and enrich the other *N-k* motifs in the same read. For example, in the following read: “…CUCCCA…”, CUCCC is responsible for SRSF3 binding. However, UCCCA will be also enriched because of the fact that about a quarter of the reads containing CUCCC have “A” immediately following the motif. To compare the binding affinity of any two co-occurring motifs, the reads where the two motifs co-occur should be excluded. In principle, a tighter binder of two motifs will have a higher R value even when the co-occurring reads are excluded. To determine which motif is responsible for the binding, we generated a correlation matrix *M* as below:

$$\left[\begin{array}{lll} M_{ii} & \cdots & M_{ij}\\ \vdots & \ddots & \vdots \\ M_{ji} & \cdots & M_{jj} \end{array}\right]$$

In this 4^5^-dimension correlation matrix, the diagonal element *M*_*ii*_ is the occurrence of motif *i* in the library and the off-diagonal element *M*_*ij*_ or *M*_*ji*_ is the occurrence that motif *i* co-occurs with motif *j* on the same reads. By this definition, the correlation matrix is symmetric as *M*_*ij*_ *= M*_*ji*_. The correlation matrix is also calculated for the negative control library and the input RNA library. The background binding by the resin was subtracted using the following formula:

$$M_{decisive,ij}=M_{sample,ij}-\left(\frac{{\left[RNA\right]}_{negative\;control}\;}{{\left[RNA\right]}_{sample}}\right)M_{negative\;control,ij}$$

$M_{sample}$ is the correlation matrix for a sample at a given protein concentration; ${\left[RNA\right]}_{negative\;control}$ is the RNA concentration of the negative control sample without protein, which is determined by Bioanalyzer; ${\left[RNA\right]}_{sample}$ is the pulled-down RNA concentration at the given protein concentration, which is determined by Bioanalyzer, and $M_{negative\;control}$ is the correlation matrix for the negative control sample without protein. The background corrected matrix ( $M_{decisive}$ ) will be used to determine which motif is responsible for binding for a given read. A problem in assigning the motif responsible for binding is that other *N*-*k* motifs that occur in the same read will also be enriched. Therefore, it is needed to determine which motif in a given read is the strongest one. For any two motifs, *i* and *j*, the stronger motif should have a higher R-value for the reads that the two motifs do not occur simultaneously. Therefore, if motif *i* is responsible for binding of a given motif *i*–bearing read, the following equation should be larger than 0 for any motif *j* among the other *N-k* motifs in the read:

$$\frac{M_{decisive,\;ii}-M_{decisive,\;ij}\;}{\sum_{i}^{4^{k}}M_{decisive,\;ii}\cdot f_{i,input}}-\frac{M_{decisive,\;jj}-M_{decisive,\;ij}\;}{\sum_{i}^{4^{k}}M_{decisive,\;ii}\cdot f_{j,input}}$$

In the above equation, subtracting off-diagonal element $M_{decisive,\;ij}$ essentially uses the reads in which motif *i* and motif *j* do not co-occur to compare the enrichment factor of the two motifs. By examining every motif for all reads (∼10,000,000), the count of binding events that motif *i* is responsible, *c*_*i*_, is calculated.

In a random RNA pool that contains all possible motifs, the dissociation constant for motif *i*, $K_{D,\;i}$, is defined as follows: $K_{D,i}=\frac{\;\left[P\right]\left[R_{i}\right]}{\left[PR_{i}\right]}$, where [*P*] is the free protein concentration, $\left[R_{i}\right]$ is the free RNA concentration for motif *i*, and $\left[PR_{i}\right]$ is the concentration of motif *i* bound with SRSF3. Considering that [*P*] is the same for all motifs, the relative $K_{D,i}^{rel}$ is proportional to $\frac{\;\left[R_{i}\right]}{\left[PR_{i}\right]}$. With the total RNA concentration for motif *i* being ${\left[R_{i}\right]}_{T}$, the above relationship can be rewritten as follows:

$$K_{D,i}^{rel}\propto \frac{{\left[R_{i}\right]}_{T}-\left[PR_{i}\right]}{\left[PR_{i}\right]}$$

The counts of NGS data are related to the RNA and complex concentration as below:

$${\left[R_{i}\right]}_{T}={\left[RNA\right]}_{input}\cdot f_{i,input}={\left[RNA\right]}_{input}\frac{M_{input,\;ii}}{\sum_{i}M_{input,\;ii}\;}$$

Where ${\left[RNA\right]}_{input}$ is the total input RNA concentration (2 μM); $f_{i,input}$ is the frequency of motif *i* in the input RNA library.

With the pulled-down RNA concentration as ${\left[RNA\right]}_{sample}$, $\left[PR_{i}\right]={\left[RNA\right]}_{sample}\frac{c_{i}}{\sum_{i}c_{i}\;}$.

Therefore,

$$K_{D\;rel,i}\propto \frac{{\left[RNA\right]}_{input}\frac{M_{input,\;ii}}{\sum_{i}M_{input,\;ii}\;}-\left({\left[RNA\right]}_{sample}\frac{c_{i}}{\sum_{i}c_{i}\;}-{\left[RNA\right]}_{negative\;control}\frac{M_{negative\;control,\;ii}}{\sum_{i}M_{negative\;control,\;ii}\;}\right)}{{\left[RNA\right]}_{sample}\frac{c_{i}}{\sum_{i}c_{i}\;}-{\left[RNA\right]}_{negative\;control}\frac{M_{negative\;control,\;ii}}{\sum_{i}M_{negative\;control,\;ii}\;}}$$

The error of relative $K_{D,i}$ is estimated as follows:

$$\sqrt{{\left(\frac{1}{c_{i}}\right)}^{2}+{\left(\frac{1}{M_{input,\;ii}}\right)}^{2}+{\left(\frac{{\left[RNA\right]}_{negative\;control}\;}{{\left[RNA\right]}_{sample}}\cdot \frac{M_{negative\;control,\;ii}}{c_{i}}\right)}^{2}+{\left(\frac{1}{M_{negative\;control,ii}}\right)}^{2}}$$

### Analysis of position contribution to specificity

The relative *K*_D_ for the 1024 motifs was grouped in such a way that in each group the motifs are only different in the position that will be analyzed. Therefore, 256 groups were created for each position. Each group contains four motifs that numerate “A”, “C”, “G”, and “U” at the position that will be analyzed. The groups that do not bind with SRSF3 were excluded from analysis. The error is estimated by $\frac{1}{\sqrt{n}}$ , where *n* is the count of the discriminating events.

## Data availability

NMR assignment data have been submitted to Biological Magnetic Resonance Data Bank (ID: 50271). The sequencing data have been deposited in SRA (BioProject ID: PRJNA688399).

## Supporting information

This article contains supporting information.

## Conflicts of interest

The authors declare that they have no conflicts of interest with the content of this article.
