# Supplementary material for: Intrinsically disordered electronegative clusters improve stability and binding specificity of RNA-binding proteins
Source: J Biol Chem. 2021 Jul 9;297(2):100945. doi: 10.1016/j.jbc.2021.100945 (PMC8348266; doi:10.1016/j.jbc.2021.100945)
Supplement: Supporting information [file mmc1.pdf]

Table S1: DNA oligomers used for RNA Bind-n-Seq

Description	Sequence (5' to 3')
T7 template	CCTTGGCACCCGAGAATTCCANNNNNNNNNNNNNNNNNNNNNNNNNNNNNNNNN NNNNNNNGATCGTCGGACTGTAGAACTCCCTATAGTGAGTCGTATTA
T7 promoter	TAATACGACTCACTATAGGG
RP1	AATGATACGGCGACCACCGAGATCTACACGTTCAGAGTTCTACAGTCCGACGATC
RPI 7	CAA GCA GAA GAC GGC ATA CGA GAT GAT CTG GTG ACT GGA GTT CCT TGG CAC CCG AGA ATT CCA
RPI 11	CAAGCAGAAGACGGCATACGAGATGTAGCCGTGACTGGAGTTCCTTGGCACCCGA GAATTCCA
RPI 12	CAAGCAGAAGACGGCATACGAGATTACAAGGTGACTGGAGTTCCTTGGCACCCGA GAATTCCA
RPI 13	CAAGCAGAAGACGGCATACGAGATTTGACTGTGACTGGAGTTCCTTGGCACCCGA GAATTCCA
RPI 14	CAAGCAGAAGACGGCATACGAGATGGAACTGTGACTGGAGTTCCTTGGCACCCGA GAATTCCA
RPI 15	CAAGCAGAAGACGGCATACGAGATTGACATGTGACTGGAGTTCCTTGGCACCCGA GAATTCCA
RPI 16	CAAGCAGAAGACGGCATACGAGATGGACGGGTGACTGGAGTTCCTTGGCACCCG AGAATTCCA
RPI 17	CAAGCAGAAGACGGCATACGAGATCTCTACGTGACTGGAGTTCCTTGGCACCCGA GAATTCCA
RT primer	GCCTTGGCACCCGAGAATTCCA

Table S2: Protein stabilities of the four Nop15 constructs at 500 mM NaCl

Nop15 constructs	ΔG (kcal·mol^{-1})	$\Delta\Delta G$ (kcal·mol^{-1})	m-value (kcal·mol^{-1}·M^{-1})	Denaturation midpoint (M)
WT	4.5 ± 0.3	-0.1	1.19 ± 0.08	3.78 ± 0.07
no-ENC	4.6 ± 0.3	0	1.23 ± 0.11	3.74 ± 0.11
no-linker	4.5 ± 0.4	-0.1	1.18 ± 0.05	3.81 ± 0.06
2xENC	4.6 ± 0.4	0.0	1.19 ± 0.07	3.87 ± 0.08

Table S3: Protein stabilities of Nop15 no-ENC at different concentrations of citrate

[Citrate] (mM)	ΔG (kcal·mol^{-1})	$\Delta\Delta G$ (kcal·mol^{-1})	m-value (kcal·mol^{-1}·M^{-1})	Denaturation midpoint (M)
0	4.6 ± 0.3	0	1.23 ± 0.11	3.74 ± 0.11
100	5.9 ± 0.3	1.3	1.38 ± 0.08	4.28 ± 0.08
200	7.3 ± 0.4	2.7	1.43 ± 0.07	5.10 ± 0.07

Table S4: Molecular dynamics simulations of three representative conformations

Conformation	ENC-contacting residues	Van der Waals interaction (kcal/mol)	Electrostatic interaction (kcal/mol)	Salt bridges*	Average distance (Å)
#1	G52, L53, A54, K65, R68, Y94, R97, R132, H133, Y134, R165, V166, L167, P168, A171, K178, Y179, K180, K181	-31	-664	E41-R165<br>E42-K179<br>E44-K181<br>D46-R132<br>D48-K178	4.7<br>3.8<br>6.8<br>5.0<br>6.4
#2	G52, L53, Y89, S90, G91, I92, K118, R121, L122, A123, R124, K127, E138, K142, V166, L167, P168, K169, A171, K172, I173, E174, Y177, K178	-38	-594	D40-K178<br>E42-R121<br>E44-R121<br>E47-K172	4.5<br>8.9<br>3.8<br>4.8
#3	G52, L53, S56, D57, D58, E59, Q60, T63, R97, N130, R132, Y134, L161, K175, K178, Y179, K180, K181	-42	-399	E41-R97<br>D43-K181<br>E44-R97<br>D46-R132	5.1<br>3.8<br>3.1<br>5.4

*Defined by the distance between the mass center of carboxylic groups in acidic residues and amino groups of Lys residues or guanidino groups in Arg residues.

Table S5: Discriminating events identified for SRSF3 using RNA Bind-n-Seq

WT 500 nM			ENC 500 nM			WT 2000 nM			ENC 2000 nM		
Site	Group	Bind when X is:	Site	Group	Bind when X is:	Site	Group	Bind when X is:	Site	Group	Bind when X is:
1	XAACG	C	1	XAAAA	C	1	XAAGT	C	1	XAACG	C
1	XAAGT	C	1	XAACG	C	1	XAATG	A	1	XAAGC	C
1	XAATG	A	1	XAAGC	C	1	XACCG	C	1	XAAGT	C
1	XACGT	C	1	XACCG	C	1	XAGCG	C	1	XAATG	C
1	XAGTA	A	1	XAGCC	C	1	XCAGT	C	1	XACCG	C
1	XCAGT	C	1	XAGCT	C	1	XCCGT	C	1	XAGCC	C
1	XCATG	C	1	XATCT	C	1	XCGTA	A	1	XAGCT	C
1	XCCGA	C	1	XCAAG	T	1	XCGTC	A	1	XCAAG	T
1	XCCGT	C	1	XCACG	C	1	XCTAG	A	1	XCAGT	C
1	XCGTA	A	1	XCATG	C	1	XGACA	C	1	XCATG	C
1	XGACA	C	1	XCCGA	C	1	XGACG	C	1	XCCGA	C
1	XTACG	C	1	XCCGG	C	1	XGACT	C	1	XCCGT	C
1	XTCAG	T	1	XCCGT	C	1	XGATA	C	1	XCCTG	C
1	XTCCG	T	1	XCGAA	C	1	XGGCA	T	1	XCGAA	A

1	XTGCC	C	1	XCGAG	C	1	XGTAA	C	1	XCGAC	A
2	AXAAG	C	1	XCGCG	C	1	XGTCT	C	1	XCGAG	C
2	AXACG	C	1	XCGCT	C	1	XTACG	C	1	XCGCT	C
2	AXATG	A	1	XCTCT	C	1	XTCAG	T	1	XCTTA	A
2	AXCCG	C	1	XCTGA	C	1	XTTGC	T	1	XCTTG	C
2	AXCTG	C	1	XCTGC	C	2	AXAAG	C	1	XGACA	C
2	AXGAA	C	1	XCTTG	C	2	AXCCG	C	1	XGCAA	C
2	AXGAC	C	1	XGAGA	C	2	AXCGT	A	1	XGCCA	C
2	CXATG	C	1	XGCCA	C	2	AXCTG	C	1	XTACG	C
2	CXCAG	C	1	XGCTC	T	2	AXGAA	C	1	XTAGC	C
2	CXCCG	C	1	XTAAA	C	2	AXGAC	C	1	XTGCC	C
2	CXCGA	C	1	XTAAT	C	2	AXGTC	C	1	XTGCT	C
2	CXCTG	C	1	XTATC	A	2	AXTAG	C	1	XTTAA	A
2	CXGAA	C	1	XTATT	A	2	CXATG	C	1	XTTAT	A
2	CXGAC	C	1	XTCTA	A	2	CXCAG	C	1	XTTCT	C
2	GXAAC	C	1	XTCTC	C	2	CXCTG	C	1	XTTTA	A
2	GXAAT	C	1	XTTCT	C	2	CXGAA	C	1	XTTTT	A
2	GXACA	C	1	XTTGC	C	2	CXGAC	C	2	AXACG	C
2	GXAGC	C	1	XTTTA	A	2	CXGCG	A	2	AXAGC	C
2	GXATA	C	1	XTTTC	A	2	CXGTA	A	2	AXCAG	C
2	GXATC	C	2	AXAAA	C	2	GXAAC	C	2	AXCCG	C
2	GXCAC	G	2	AXAGC	C	2	GXAAT	C	2	AXGAA	C
2	GXCTC	C	2	AXCAG	C	2	GXACA	C	2	AXGAC	C
2	GXCTT	C	2	AXCCG	C	2	GXACT	C	2	AXGCC	C
2	GXGCA	C	2	AXCTC	C	2	GXATA	C	2	AXTGC	A
2	GXGC C	C	2	AXCTG	C	2	GXATC	C	2	CXCAG	C
2	GXTAA	C	2	AXCTT	C	2	GXCAC	G	2	CXCGA	C
2	GXTAC	C	2	AXTTT	A	2	GXCCA	C	2	CXCGT	C
2	GXTCC	C	2	CXAGA	G	2	GXCTA	C	2	CXCTG	C
2	GXTTT	T	2	CXATG	C	2	GXCTC	C	2	CXGAG	C
2	TXAAG	C	2	CXCAG	C	2	GXCTT	C	2	CXTTA	A
2	TXAGC	C	2	CXCGA	C	2	GXGCA	C	2	CXTTG	C
2	TXCTG	C	2	CXCGG	C	2	GXGCC	C	2	CXTTT	A
2	TXGAC	C	2	CXCGT	C	2	GXTAA	C	2	GXAAA	C
3	AAXAG	C	2	CXCTG	C	2	GXTAC	C	2	GXAAC	C
3	AAXTG	A	2	CXGAA	C	2	GXTCC	C	2	GXACA	C
3	ACXTG	C	2	CXGAG	C	2	GXTGC	C	2	GXACT	C
3	AGXAC	C	2	CXGCG	C	2	GXTTT	T	2	GXAGC	C
3	AGXAT	C	2	CXGGC	T	2	TXAAG	C	2	GXATC	C
3	AGXCC	C	2	CXTGA	C	2	TXACG	C	2	GXCAC	G
3	AGXCT	C	2	CXTTA	A	2	TXCGA	A	2	GXCCC	C
3	AGXGC	C	2	CXTTG	C	2	TXCGT	A	2	GXCTT	C

3	AGXTA	C	2	CXTTT	A	2	TXCTG	C	2	GXGCA	C
3	AGXTC	C	2	GXACA	C	2	TXGAC	C	2	GXGCC	C
3	AGXTT	C	2	GXACT	C	3	AAXAG	C	2	GXTAC	C
3	ATXGC	A	2	GXAGC	C	3	AAXCG	A	2	GXTCC	C
3	CAXCG	A	2	GXATC	C	3	AAXGA	C	2	GXTTC	C
3	CCXAG	C	2	GXCAC	G	3	AAXGT	C	2	TXAAA	C
3	CCXGA	C	2	GXCAT	G	3	AAXTG	A	2	TXAAG	C
3	CGXAC	C	2	GXCCC	C	3	ACXGA	C	2	TXAAT	C
3	CGXAT	C	2	GXCCG	C	3	AGXAC	C	2	TXAGC	C
3	CGXCC	C	2	GXGCC	C	3	AGXAT	C	2	TXATC	C
3	CGXCT	C	2	GXTAC	C	3	AGXCA	C	2	TXCCG	C
3	CGXG C	C	2	GXTCC	C	3	AGXCC	C	2	TXGCA	G
3	CGXTA	C	2	TXAAA	C	3	AGXCT	C	2	TXTAA	C
3	CGXTC	C	2	TXAAG	C	3	AGXGC	C	3	ACXAG	C
3	CGXTT	C	2	TXAAT	C	3	AGXTA	C	3	AGXAC	C
3	CTXCG	A	2	TXACA	C	3	AGXTC	C	3	AGXAT	C
3	GAXAA	A	2	TXAGC	C	3	AGXTT	C	3	AGXCA	G
3	GCXAT	A	2	TXATC	C	3	ATXGC	A	3	AGXCC	C
3	GCXG C	A	2	TXCCG	C	3	CAXGA	C	3	AGXCT	C
3	GCXTA	A	2	TXCTA	A	3	CCXAG	C	3	ATXCT	A
3	GCXTT	C	2	TXCTT	A	3	CCXGA	C	3	CAXGT	A
3	GGXAC	C	2	TXGCA	G	3	CGXAC	C	3	CAXTG	A
3	GGXC C	C	2	TXGGC	T	3	CGXAT	C	3	CCXGA	C
3	GTXCC	A	3	AAXGC	T	3	CGXCC	C	3	CGXAA	C
3	GTXTC	T	3	AAXTC	A	3	CGXCG	A	3	CGXAC	C
3	GTXTT	T	3	ACXAG	C	3	CGXGC	C	3	CGXAT	C
3	TAXAG	C	3	ACXCG	C	3	CGXTC	C	3	CGXCC	C
3	TCXCG	C	3	ACXGC	A	3	CGXTT	C	3	CGXCT	C
3	TCXTG	C	3	ACXTG	C	3	GAXAA	A	3	CGXGC	C
3	TGXAA	C	3	AGXAC	C	3	GCXAT	A	3	CGXTC	C
3	TGXAC	C	3	AGXAT	C	3	GCXCT	A	3	CTXCG	A
3	TGXAT	C	3	AGXCA	G	3	GCXGC	T	3	CTXGC	A
3	TGXCA	G	3	AGXCC	C	3	GCXTT	C	3	GCXAA	A
3	TGXCC	C	3	AGXGC	T	3	GGXAC	C	3	GCXCT	A
3	TGXCT	C	3	ATXAA	C	3	GGXCC	C	3	GCXGC	A
3	TGXTC	C	3	ATXAT	C	3	GTXCC	A	3	GCXTT	C
3	TGXTT	C	3	ATXTT	A	3	GTXTC	T	3	GGXAC	C
3	TTXAG	C	3	CCXGG	C	3	GTXTT	T	3	GTXCC	A
3	TTXCG	C	3	CCXGT	C	3	TAXAG	C	3	TAXAA	C
3	TTXGC	T	3	CGXAC	C	3	TAXGA	C	3	TAXAT	C
4	AAAXG	T	3	CGXAT	C	3	TAXGT	C	3	TAXCT	C

4	AACXG	A	3	CGXCA	C	3	TCXTG	C	3	TAXTA	C
4	AAGXC	C	3	CGXCC	C	3	TGXAA	C	3	TAXTT	C
4	AAGXT	C	3	CGXCT	C	3	TGXAC	C	3	TCXAG	A
4	ACGXT	C	3	CGXGA	A	3	TGXAT	C	3	TCXCG	C
4	AGAXA	A	3	CGXGC	C	3	TGXCA	G	3	TCXTA	C
4	AGGXA	C	3	CTXAA	A	3	TGXCC	C	3	TGXAC	C
4	ATGXA	C	3	CTXAT	A	3	TGXCT	C	3	TGXCA	G
4	ATGXT	C	3	CTXTC	C	3	TGXGC	C	3	TGXCC	C
4	CAAXG	C	3	GAXCA	G	3	TGXTA	C	3	TGXCT	C
4	CAGXA	C	3	GCXAC	T	3	TGXTC	C	3	TGXGC	C
4	CAGXC	C	3	GCXCG	C	3	TGXTT	C	3	TGXTC	C
4	CAGXT	C	3	GCXCT	A	3	TTXAG	C	3	TTXAA	C
4	CCGXT	C	3	GCXGC	A	3	TTXCG	C	3	TTXAT	C
4	CTAXG	C	3	GCXTC	A	4	AACXG	A	3	TTXTA	C
4	CTGXA	C	3	GGXAC	C	4	AAGXC	C	3	TTXTC	T
4	CTGXC	C	3	GGXAT	C	4	AAGXT	C	3	TTXTT	C
4	CTGXT	C	3	GTXCA	G	4	ACGXT	C	4	AAGXA	C
4	GAAXA	A	3	GTXCC	A	4	ACTXG	A	4	ACAXG	C
4	GCAXT	A	3	TAXAA	C	4	AGAXA	A	4	AGGXA	C
4	GCCXT	T	3	TAXAT	C	4	ATGXA	C	4	ATGXA	C
4	GCGXA	C	3	TAXCA	C	4	ATGXC	C	4	CACXG	C
4	GCGX C	C	3	TAXCT	C	4	ATGXT	C	4	CAGXA	C
4	GCTXA	A	3	TAXTA	C	4	CAAXG	C	4	CAGXC	C
4	GTAXC	C	3	TAXTT	C	4	CACXG	C	4	CAGXT	C
4	GTTXC	T	3	TCXAG	A	4	CAGXC	C	4	CCGXA	C
4	GTTXT	T	3	TCXCG	C	4	CAGXG	C	4	CCGXC	C
4	TACXG	A	3	TCXCT	C	4	CAGXT	C	4	CCGXG	A
4	TAGXA	C	3	TGXAC	C	4	CCGXT	C	4	CCGXT	C
4	TCAXG	A	3	TGXAT	C	4	CGAXG	C	4	CCTXG	T
4	TCGXC	A	3	TGXCA	G	4	CGAXT	C	4	CGAXA	C
4	TGCXA	A	3	TGXCC	C	4	CGTXA	A	4	CTAXG	C
4	TGGXA	C	3	TGXCT	C	4	CGTXT	C	4	CTGXA	C
5	AAAGX	C	3	TGXTC	C	4	CTAXG	C	4	CTGXC	C
5	AACGX	C	3	TTXAA	C	4	CTCXG	C	4	CTGXT	C
5	AAGTX	A	3	TTXAT	C	4	CTGXA	C	4	CTTXA	C
5	AATGX	C	3	TTXCA	C	4	CTGXC	C	4	CTTXT	C
5	ACAGX	C	3	TTXCT	C	4	CTGXT	C	4	GCAXT	C
5	ACCGX	C	3	TTXGC	G	4	GAAXA	A	4	GCCXC	C
5	ACGTX	A	4	AAGXA	C	4	GCCXT	T	4	GCCXT	T
5	ACTGX	C	4	ACGXA	C	4	GCGXA	C	4	GCGXA	C
5	AGAAX	A	4	ACGXC	C	4	GCGXC	C	4	GCGXC	C
5	AGCGX	C	4	ACTXT	A	4	GCTXA	A	4	GGCXC	A

5	AGGCX	A	4	AGCXT	A	4	GTAXC	C	4	GTAXC	C
5	ATAGX	C	4	AGGXA	C	4	GTTXC	T	4	TAAXA	C
5	CATGX	C	4	AGTXC	G	4	GTTXT	T	4	TATXA	C
5	CCTGX	C	4	ATAXA	C	4	TACXG	A	4	TCAXG	A
5	CGAAX	A	4	ATGXA	C	4	TAGXA	C	4	TCCXG	C
5	CGACX	A	4	ATGXC	C	4	TCGXC	A	4	TGCXT	C
5	CGCG X	C	4	CAAXG	C	4	TGGXA	C	4	TGGXA	C
5	CTAGX	C	4	CACXG	C	5	AAAGX	C	4	TTAXA	C
5	CTTGX	C	4	CAGXA	C	5	AAGTX	A	4	TTAXT	C
5	GAAAX	A	4	CAGXC	C	5	AATGX	C	4	TTTXA	C
5	GCAGX	C	4	CAGXT	C	5	ACAGX	C	5	AACGX	C
5	GCCCX	C	4	CCGXC	C	5	ACTGX	C	5	AAGCX	A
5	GCTCX	C	4	CCGXT	C	5	AGAAX	A	5	AATGX	C
5	GCTTX	C	4	CCTXG	T	5	AGCGX	C	5	ACAGX	C
5	GGCAX	C	4	CGAXA	G	5	ATAGX	C	5	ACCGX	C
5	GGCC X	C	4	CGCXA	C	5	CAGTX	A	5	AGGCX	A
5	GTACX	C	4	CTCXA	C	5	CATGX	C	5	ATGCX	A
5	TACGX	C	4	CTCXT	C	5	CCTGX	C	5	CACGX	C
5	TAGCX	A	4	CTGXA	C	5	CGAAX	A	5	CATGX	C
5	TATGX	C	4	CTTXA	C	5	CGATX	A	5	CCGAX	G
5	TCAGX	C	4	CTTXT	C	5	CGCGX	C	5	CCTGX	C
5	TCCGX	C	4	GAGXA	C	5	CGTAX	A	5	CGACX	A
5	TCGAX	C	4	GCAXA	C	5	CGTCX	T	5	CGCGX	C
5	TCTGX	C	4	GCAXT	C	5	CTAGX	C	5	CGCTX	C
5	TGGCX	A	4	GCCXC	C	5	CTCGX	C	5	CTAGX	C
5	TTTGX	C	4	GCCXG	C	5	GAAAX	A	5	CTTAX	C
			4	GCGXA	C	5	GCTCX	C	5	GCAGX	C
			4	GCGXC	C	5	GCTGX	C	5	GCATX	C
			4	GGCXC	A	5	GCTTX	C	5	GCCCX	C
			4	GGCXT	A	5	GGCAX	C	5	GCCTX	T
			4	GTAXC	C	5	GGCCX	C	5	GCTAX	C
			4	GTGXA	C	5	GTACX	C	5	GCTCX	C
			4	TCAXG	A	5	TAAGX	C	5	GCTTX	C
			4	TCAXT	A	5	TAGCX	A	5	GGCAX	C
			4	TCCXG	C	5	TATGX	C	5	GTACX	C
			4	TGGXA	C	5	TCAGX	C	5	TAAAX	C
			4	TGTXC	G	5	TCCGX	C	5	TACGX	C
			4	TTAXC	C	5	TCGAX	C	5	TATAX	C
			4	TTGXC	G	5	TCTGX	C	5	TATGX	C
			5	AACTX	A	5	TGCGX	C	5	TATTX	C
			5	AAGCX	A	5	TGGCX	A	5	TCAGX	C
			5	AATGX	C	5	TTCGX	C	5	TCATX	C

			5	ACAGX	C	5	TTTGX	C	5	TCCGX	C
			5	ACTTX	C				5	TCTGX	C
			5	AGCCX	C				5	TCTTX	C
			5	AGGCX	A				5	TGCAX	C
			5	AGTGX	C				5	TGCGX	C
			5	ATAAX	C				5	TGCTX	C
			5	ATCTX	A				5	TGGCX	A
			5	ATTAX	C				5	TTAAX	C
			5	CAAGX	C				5	TTTAX	C
			5	CACGX	C				5	TTTTX	C
			5	CATGX	C						
			5	CCAGX	C						
			5	CGAGX	A						
			5	CGCGX	C						
			5	CTCAX	C						
			5	CTCTX	C						
			5	CTGGX	C						
			5	CTTGX	C						
			5	GAGCX	A						
			5	GCAGX	C						
			5	GCATX	C						
			5	GCTAX	C						
			5	GCTCX	C						
			5	GTACX	C						
			5	GTGCX	A						
			5	TAAAX	C						
			5	TAACX	C						
			5	TACGX	C						
			5	TATAX	C						
			5	TATCX	C						
			5	TATGX	C						
			5	TATTX	C						
			5	TCAGX	C						
			5	TCATX	C						
			5	TCCGX	C						
			5	TCCTX	C						
			5	TCTAX	C						
			5	TCTCX	C						
			5	TCTTX	C						
			5	TGCGX	C						
			5	TGCTX	C						
			5	TGGCX	A						
			5	TGTGX	C						

			5	TTACX	C						
			5	TTGGX	C						
			5	TTTAX	C						
			5	TTTCX	C						

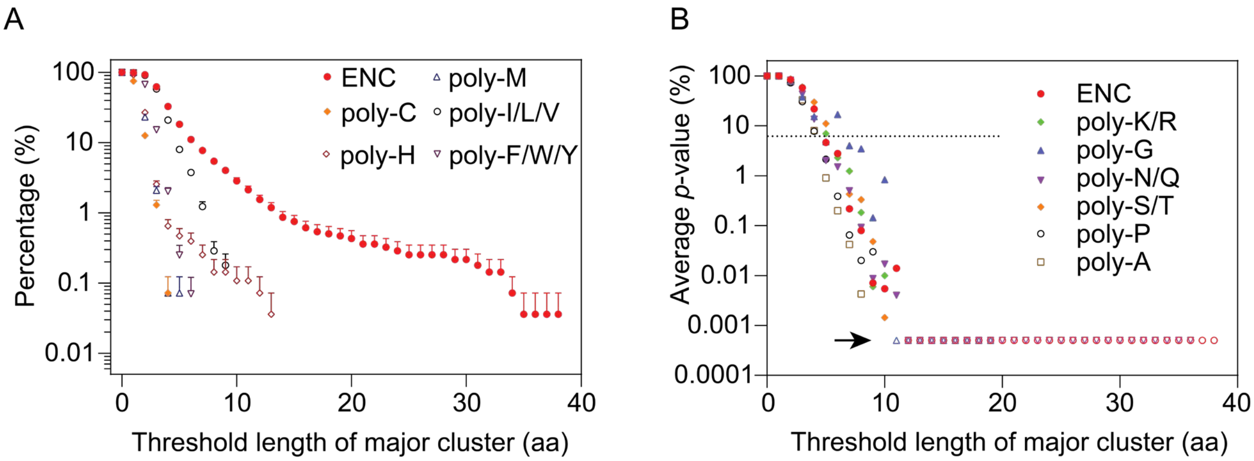

Figure S1: Repetitive cluster occurrence and *p*-value analysis. (A) Occurrence of poly-C, poly-H, poly-M, poly-I/L/V, and poly-F/W/Y compared with ENC. For clarity, only top halves of error bars (┬) are shown. (B) Average *p*-values for consecutive major clusters. The *p*-values for the clusters whose occurrence were not found in 100,000 Monte Carlo simulations were lower than 0.001% and denoted by a black arrow. The dash line indicates 5%.

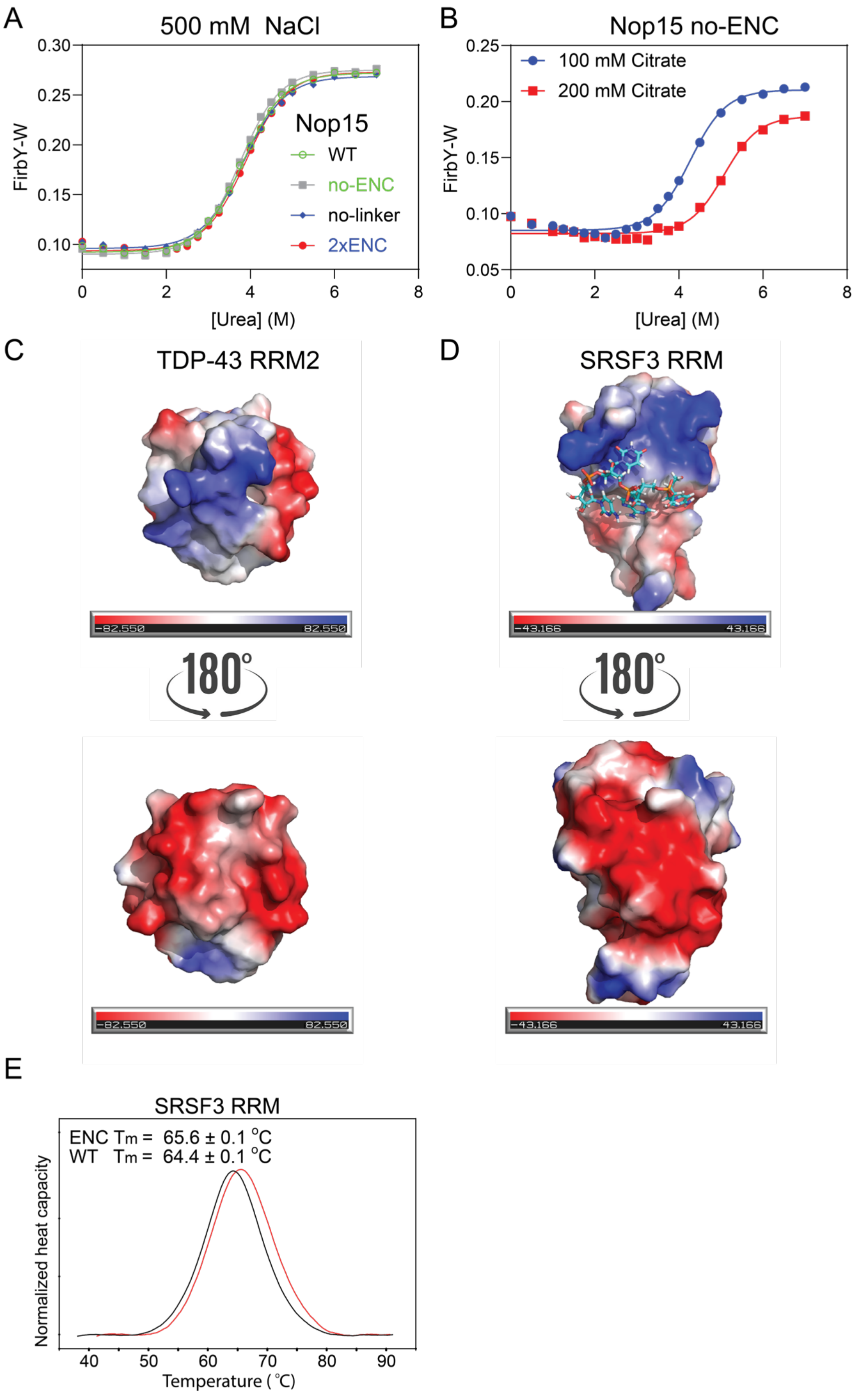

Figure S2: (A) Effects of the ENC on Nop15 unfolding thermodynamics in the presence of 500 mM NaCl. (B) Effects of citrate on the stability of Nop15 no-ENC at different concentrations. The protein stability was measured using FirbY-W for A and B. (C) Surface electrostatic potential of TDP-43 RRM2. (D) Surface electrostatic potential of SRSF3 RRM. The RNA is shown as cyan sticks. The unit of the potential bar is $K_b T/e$, where K_b is the Boltzmann constant; T is temperature in the Kelvin scale and e is the elementary charge. (E) The normalized DSC melting curves for the wild-type and ENC-engineered SRSF3 RRM. The error was estimated from individual measurements on proteins of three individual preparations.

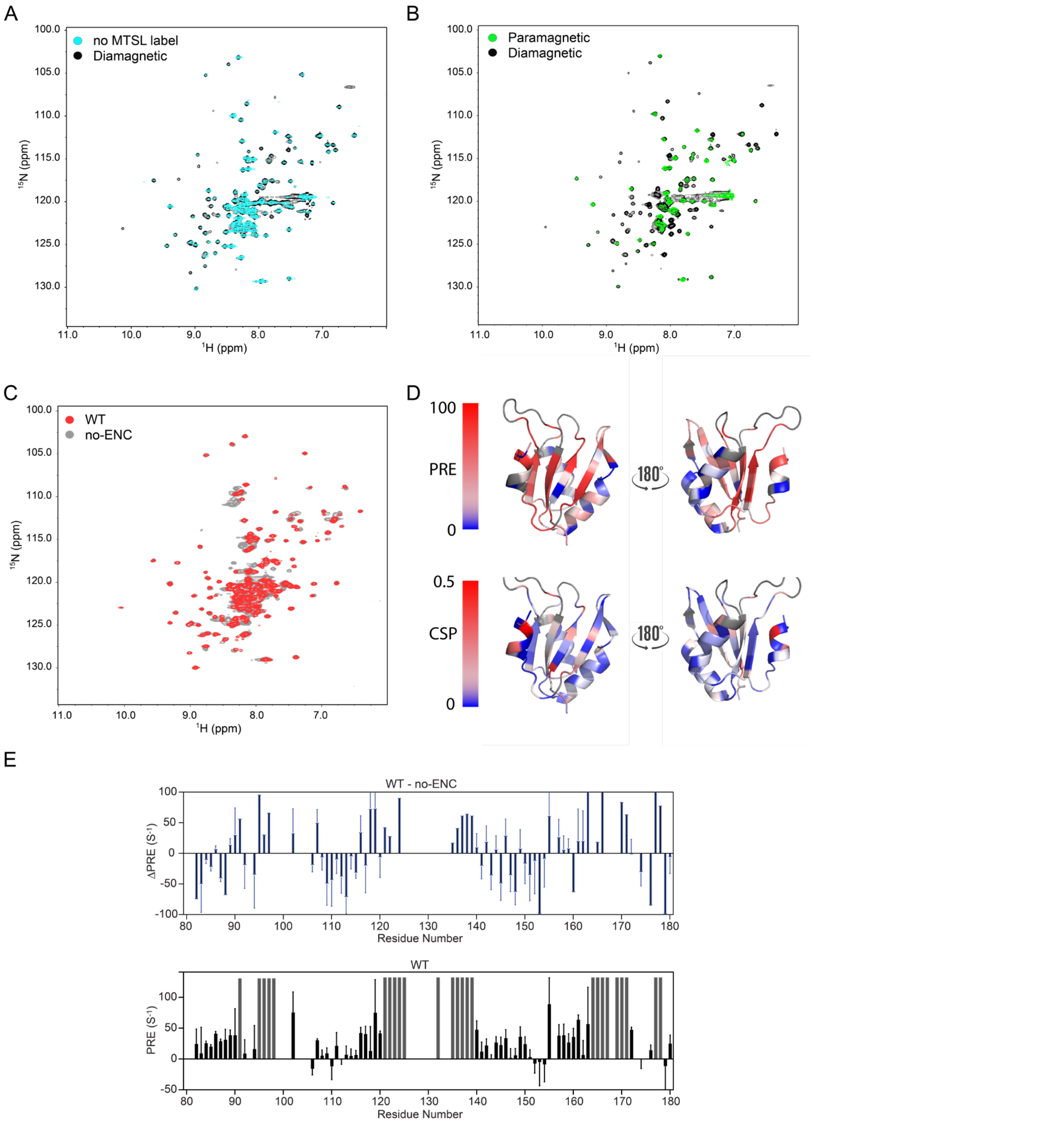

Figure S3: (A) ^{15}N-HSQC spectra of Nop15 with and without MTSL label are shown in black and cyan, respectively. (B) ^{15}N-HSQC spectra of Nop15 collected in the para- and dia-magnetic states are shown in green and black, respectively. (C) Chemical shift perturbation (CSP) analysis of intramolecular interaction between the Nop15 ENC and RRM. CSP values were obtained by comparing the chemical shifts of no-ENC and WT Nop15 using the formula $|\delta^{1}H|+0.1^{*}|\ \delta^{15}N|$. (D) Plot of PRE (top) and CSP (bottom) values onto the Nop15 RRM. The units for PRE and CSP scale bars are s^{-1} and ppm, respectively. (E) PRE difference (ΔPRE) between the wild-type and no-ENC Nop15 is shown in the top panel. The PRE values for wild-type Nop15 was shown in bottom for a convenient direct comparison. Gray bars indicate the bleached residues, whose amide resonances disappear due to close proximity to MTSL. To calculate PRE difference, PRE values for bleached residues are assumed to be 100 s^{-1}, which is an order of magnitude lower than the real values. The error was estimated by $[(PRE_{WT}^{error})^2 + (PRE_{no\text{-}ENC}^{error})^2]^{1/2}$ for the residues that are not bleached.

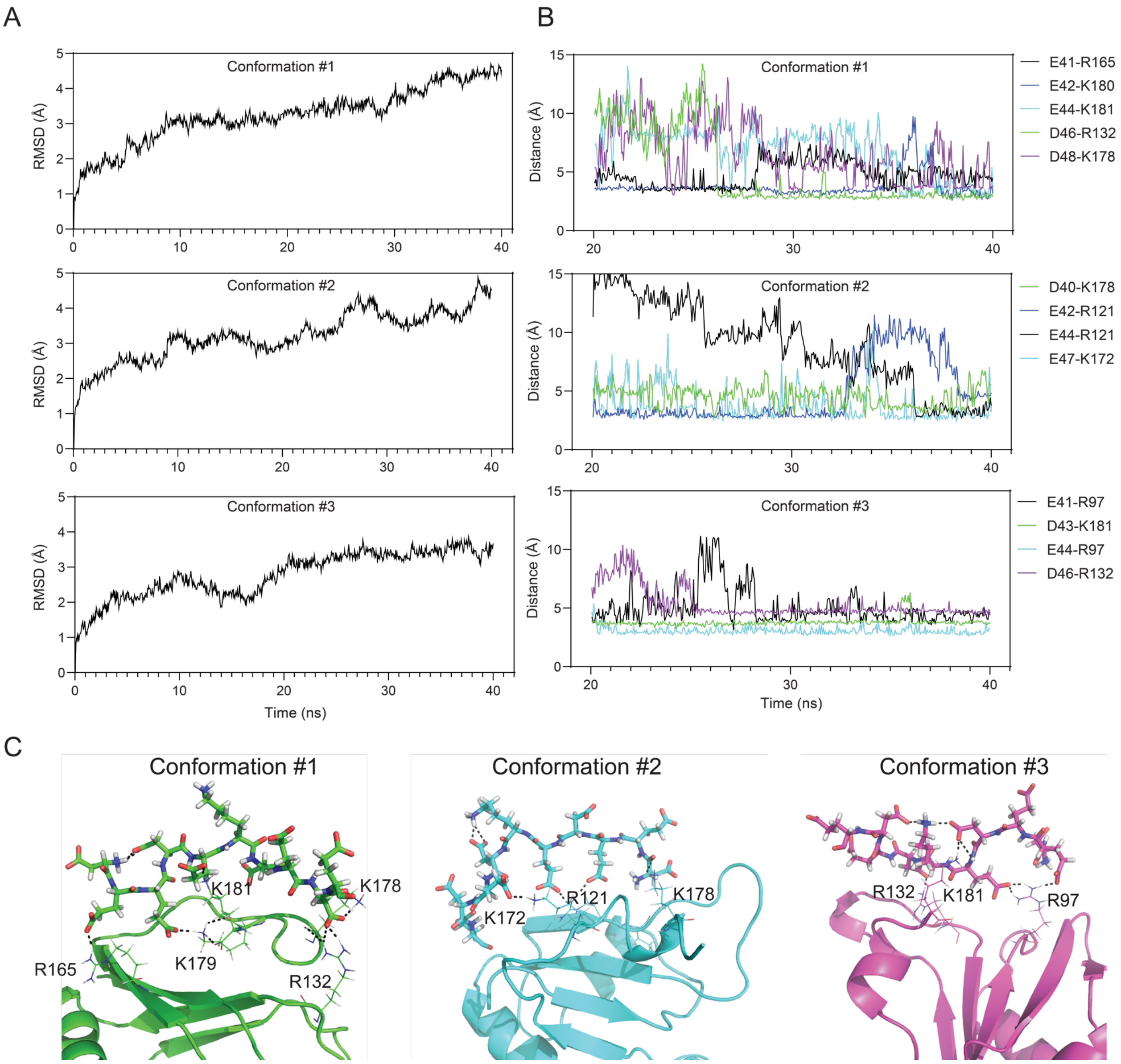

Figure S4: MD simulation of the intramolecular interaction between the Nop15 ENC and RRM. (A) MD simulation trajectories for three representative conformations. (B) MD simulation trajectories of salt bridges in the three representative conformations. (C) Three representative conformations of the molecular dynamic simulation. The Nop15 with ENC in three conformations are shown in cartoon and colored green, cyan and purple, respectively. H-bonds and salt bridges for the three conformations are denoted by black dotted lines. The RRM residues involved in ENC interaction are shown in lines and are labeled in the figure.

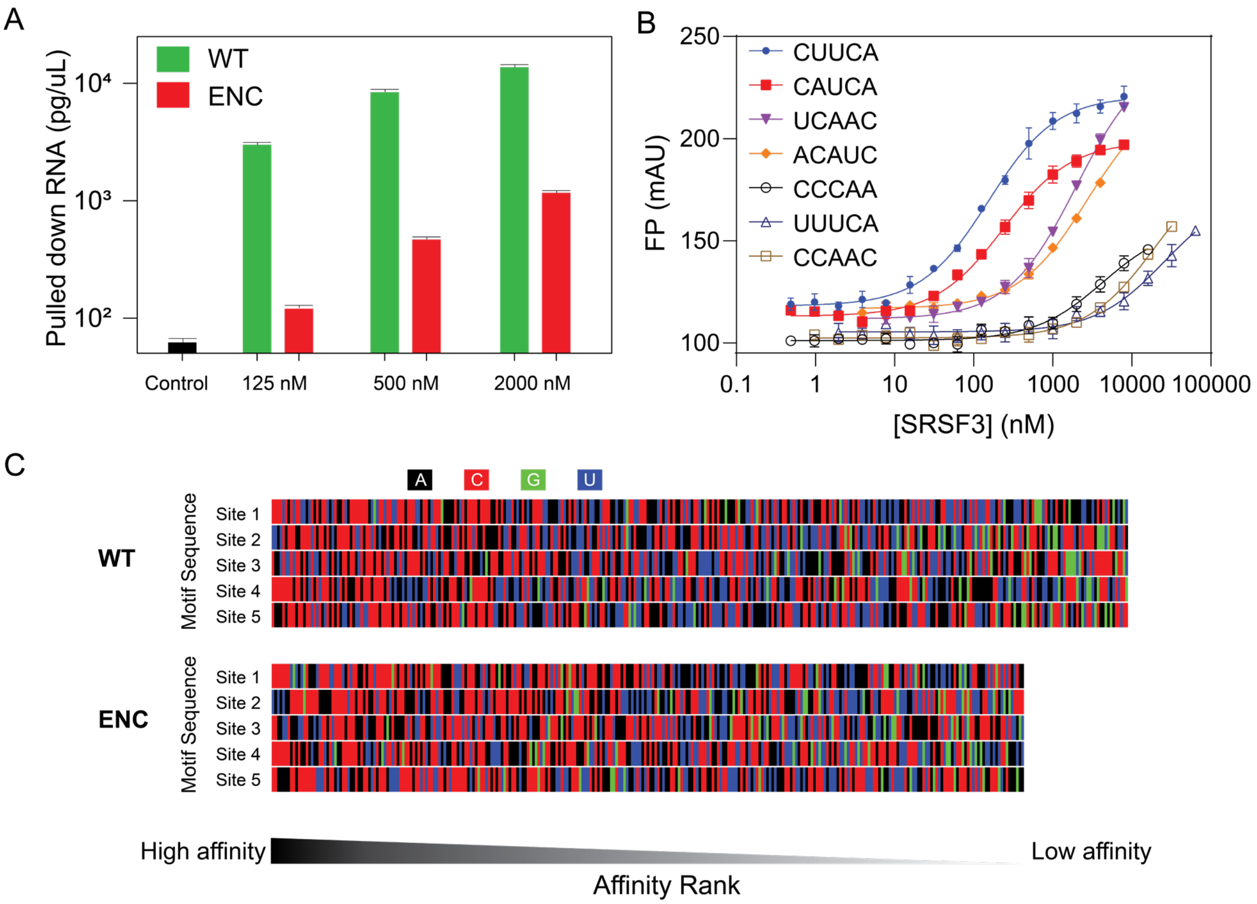

Figure S5: An engineered ENC increases RNA-binding specificity of SRSF3. (A) The amount of RNA pulled down by the WT and ENC-mutant SRSF3 at different concentrations. (B) FP binding profiles for SRSF3 with seven different 5-mer RNA motifs. Errors were estimated from three individual measurements. The RNA molecules have a fluorescein label at the 5' ends. (C) Sequence patterns of RNA pulled down at 2000 nM wild-type and mutant SRSF3. The types of nucleotides at each site are shown by denoted colors.

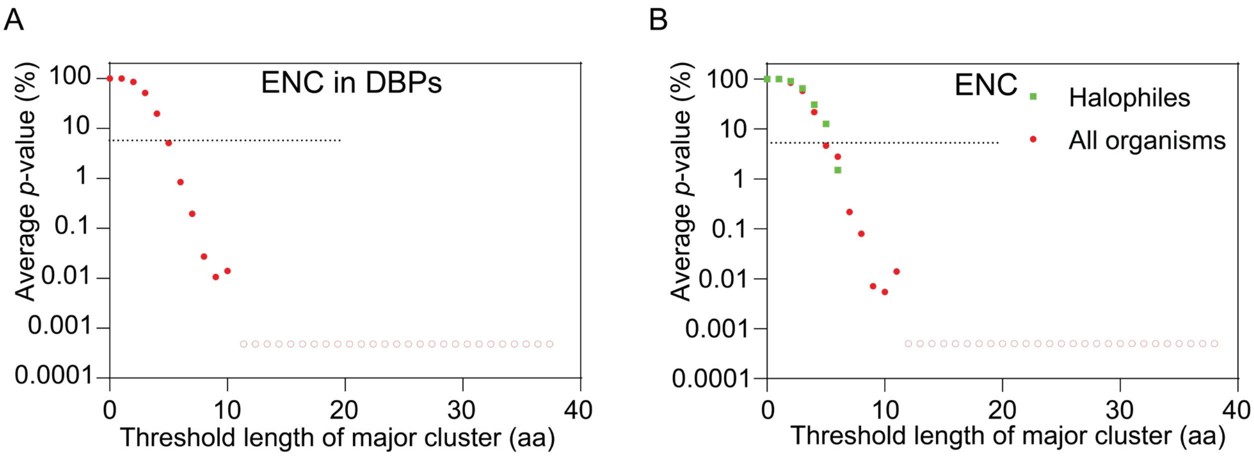

Figure S6: (A) Average *p*-values for ENCs in DNA-binding proteins. (B) Average *p*-values for ENCs in halophiles. The *p*-values for the ENCs whose occurrence were not found in 100,000 Monte Carlo simulations were lower than 0.001% and shown by open symbols. The dash line indicates 5%.
